# A general algorithm for error-in-variables regression modelling using Monte Carlo expectation maximization

**DOI:** 10.1371/journal.pone.0283798

**Published:** 2023-04-03

**Authors:** Jakub Stoklosa, Wen-Han Hwang, David I. Warton

**Affiliations:** 1 The University of New South Wales and Evolution & Ecology Research Centre, Sydney, New South Wales, Australia; 2 Institute of Statistics, National Tsing Hua University, Hsinchu, Taiwan; University of Oviedo: Universidad de Oviedo, SPAIN

## Abstract

In regression modelling, measurement error models are often needed to correct for uncertainty arising from measurements of covariates/predictor variables. The literature on measurement error (or errors-in-variables) modelling is plentiful, however, general algorithms and software for maximum likelihood estimation of models with measurement error are not as readily available, in a form that they can be used by applied researchers without relatively advanced statistical expertise. In this study, we develop a novel algorithm for measurement error modelling, which could in principle take *any* regression model fitted by maximum likelihood, or penalised likelihood, and extend it to account for uncertainty in covariates. This is achieved by exploiting an interesting property of the Monte Carlo Expectation-Maximization (MCEM) algorithm, namely that it can be expressed as an iteratively reweighted maximisation of complete data likelihoods (formed by imputing the missing values). Thus we can take any regression model for which we have an algorithm for (penalised) likelihood estimation when covariates are error-free, nest it within our proposed iteratively reweighted MCEM algorithm, and thus account for uncertainty in covariates. The approach is demonstrated on examples involving generalized linear models, point process models, generalized additive models and capture–recapture models. Because the proposed method uses maximum (penalised) likelihood, it inherits advantageous optimality and inferential properties, as illustrated by simulation. We also study the model robustness of some violations in predictor distributional assumptions. Software is provided as the refitME package on R, whose key function behaves like a refit() function, taking a fitted regression model object and re-fitting with a pre-specified amount of measurement error.

## Introduction

Measurement error (or error-in-variables) models are often needed to correct for uncertainty arising from measurements of covariates (*e.g*., imprecise measurements of body weights for patients in a clinical study, or using noisy instruments to measure temperature in field studies) to avoid the “double-whammy” of bias and inefficiency [1]. The literature on measurement error modelling is plentiful, for example, [1] reviews a number of well-known measurement methods, these include: regression (and refined regression) calibration, simulation extrapolation (SIMEX), corrected and conditional score, and Bayesian hierarchical models. Some recent developments include: inference for high dimensional measurement error models *e.g*., the lasso [2–4], matrix-variate measurement error models *e.g*., for image analysis [5], machine learning algorithms [6] and graphical models [7]. Our study focuses on structural methods with classical measurement error structures where measurements are not replicated, so methods such as [8], for example, are unavailable.

General maximum likelihood algorithms for fitting models with measurement error are not readily available, in a form that they can be used by applied researchers without relatively advanced statistical expertise. A difficulty is that in many situations (*e.g*., for non-Gaussian responses), introducing measurement error to a model means that the likelihood no longer has a closed form, significantly complicating estimation. While a Bayesian hierarchical model could readily be constructed and fitted using generic Monte Carlo algorithms [9], this would need to be done in a case-specific way that requires some expertise in statistical programming. Another way to use Monte Carlo (MC) integration techniques [10] in measurement error modelling is to combine them with an Expectation-Maximization algorithm, because it often simplifies the optimization step. Solutions to date have however tended to be case-specific, taking a particular class of models and extending them to account for measurement error, such as [11–13]. An alternative, outside the likelihood-based framework, is simulation-extrapolation [14, 15].

The software landscape for measurement error modelling is similarly piecemeal. The most comprehensive in terms of functionality is the simex package [15, 16], recently extended to be able to fit measurement error models to Generalized Additive Models (GAMs) [17], survival analysis and mixed models. Otherwise, software tends to be specific to GAMs particular problems including linear models [18], correlated errors [19] and high-dimensional regression [3]. An example problem for which there are currently no software options is measurement error modelling of Point process models (PPMs). Point process models are being increasingly widely used in ecology to predict species distribution from sighting events [20, 21], and covariates commonly come with uncertainty. Several measurement error methods have been developed for GAMs [22–24], however these methods were only established for Gaussian responses and without generally available software implementations. Capture–recapture models are another important type of model in ecology, for which better measurement error modelling techniques are needed. Some capture–recapture models have been extended to handle measurement error as a function of linear predictors [25], but not previously in the non-linear (non-parametric) case.

A further issue arising from lack of generality is that when new models are developed that are intended for error-free covariates, as is happening at an increasingly rapid pace, measurement error extensions would need to be developed as a distinct subsequent step. Thus, the measurement error modelling literature is always playing “catch-up”. A generic measurement error modelling algorithm, which could act as a “wrapper” around any given model-fitting algorithm, would address this issue.

In this study, we introduce the refitME software for maximum likelihood estimation of measurement error models using a novel adaptation of the Monte-Carlo EM algorithm (MCEM) [26, 27]. This algorithm can extend any model to handle measurement error in covariates, given an algorithm that could fit the model in the absence of measurement error via maximum (penalised) likelihood estimation, although our current implementation of refitME is limited to fixed effects modelling of responses taking a common distribution from the exponential family. MCEM algorithms have previously been applied in the regression setting, especially to missing data problems [28] or hierarchical modelling [29], but less so to measurement error modelling. Our algorithm differs from conventional applications of the MCEM algorithm as it is implemented in an iteratively reweighted fashion. Specifically, we simulate multiple Monte Carlo realisations of the true values of covariates, and refit the original modelling algorithm to this complete dataset, with (importance sampling) weights that are updated each iteration. Thus, the approach is very general, and can be implemented to any problem for which we already have an algorithm for maximum (penalised) likelihood estimation in the absence of measurement error. Like other maximum likelihood algorithms to measurement error modelling [11, 30], also see Chapters 6 and 7 of [1], our approach requires knowledge of the distribution of the measurement errors and the form of the distribution for the error-free covariates. Our approach sacrifices some computational efficiency in order to gain generality—as we illustrate, the method can readily be used for applications as diverse as point process modelling, non-parametric regression, and capture–recapture modelling with non-linear response to predictors.

Notation, estimation details and algorithm properties for MCEM are given in the Materials and methods section. We conduct simulations to examine bias, efficiency, confidence interval coverage, prediction properties and robustness to violations in model assumptions, and then demonstrate the use of MCEM on several real-data examples where covariates are subject to measurement error in the Results section. We then present the refitME R-package which implements MCEM algorithm in the Software section. Some final conclusions are presented in the Discussion section.

## Materials and methods

### Notation and measurement error models

Let ***Y*** = (*Y*_1_, …, *Y*_*n*_) be an *n*-vector of i.i.d. responses with distribution *f*_*Y*_ and denote *y*_*i*_ as the observed realization of *Y*_*i*_ for *i* = 1, …, *n*. Let *p* be the number of error contaminated covariates and ***X*** = [***X***_1_ ⋯ ***X***_*n*_]^*T*^ be an *n* × *p* matrix of true covariate values where ***X***_*i*_ = (*X*_*i,1*_, …, *X*_*i,p*_) is a *p*-vector. We assume a parametric model for the continuous random variable ***X*** which in practice is usually assumed to follow the multivariate normal distribution, denoted by *f*_*X*_. Let ***β*** be the parameter vector associated with ***X***. We consider a general regression model to estimate ***β*** where we assume a univariate response and use complete-case data—*i.e*., we assume there is no missingness in the response and covariate. A common example is a generalized linear model, for which $E\left(Y|X\right)=g^{-1}\left(X\beta \right)$ where *g*(⋅) is the link function.

A classical measurement error model [1] assumes an additive error structure for ***X***, such that ***W*** = ***X*** + ***U*** with ***X*** and ***U*** being independent of each other where ***W*** are the error-contaminated covariate values and ***U*** are the measurement errors with distribution *f*_*U*_. We assume that *U* follows the normal distribution, such that $U_{i,k}∼N\left(0,\sigma_{u,k}^{2}\right)$ are i.i.d. for all 1 ≤ *i* ≤ *n* and 1 ≤ *k* ≤ *p* with $\sigma_{u,k}^{2}$ being known variances for the *k*th corresponding error-contaminated covariate. The measurement error variance can be estimated using repeated measures, or sometimes, with validation data *e.g*., see [31]. For any covariates measured with error, we assume these variables are continuous. In contrast, no distributional assumptions would be required for predictors that were measured without error. Finally, denote ***y***, ***x*** and ***w*** as realizations of ***Y***, ***X*** and ***W***, respectively.

### Monte-Carlo (MC) EM algorithm

Our objective is to maximize the observed log-likelihood *ℓ*(***β***) = log {*f*_*Y*_(***y***, ***w***; ***β***)} w.r.t. ***β***; however, ***X*** is an unobserved (latent) variable that must be considered in the target function. One approach to obtaining a maximum likelihood estimate of ***β***, or a penalised likelihood estimate, is via an EM-algorithm [32]. We follow [33] and use similar notation when referring to joint, marginal and conditional density functions throughout. In particular, we use *f*_*X*∣*W*,*Y*_ and $E_{X|W,Y}$ to denote the conditional density and expectation, respectively, of *X* given the observed data *y* and *w*. Assuming that *f*_*X*_ is known, the EM algorithm iterates between a calculation of the expected complete-data likelihood (“*E*-step”):

$$\begin{array}{cc} Q(\beta |{\hat{\beta }}^{\left[t\right]}) & =\underset{X|W,Y}{E}[\text{log}\;\{f_{Y}\left(y,w,x;\beta \right)\}]-h\left(\beta \right)\\ & =\int \;\text{log}\;\{f_{Y}\left(y|x;\beta \right)f_{W}\left(w|x\right)f_{X}\left(x|{\hat{\beta }}^{\left[t\right]}\right)\}f_{X|W,Y}\left(x\right)dx-h\left(\beta \right), \end{array}$$ (1)

and a maximization of $Q(\beta |{\hat{\beta }}^{\left[t\right]})$ w.r.t. ***β*** (“*M*-step”), where ${\hat{\beta }}^{\left[t\right]}$ denotes the *t*th iteration and ${\hat{\beta }}^{\left[t+1\right]}=\underset{\beta }{\text{arg}\;\text{max}}\;Q\left(\beta |{\hat{\beta }}^{\left[t\right]}\right)$. An arbitrary penalty term *h*(***β***) has been included in Eq (1) to indicate that this approach applies to penalised likelihood as well as to maximum likelihood (*e.g*., GAMs, see Example 2: Generalized additive models). EM-type algorithms are often useful because the *M*-step can often be expressed in a relatively simple form.

For higher dimensions and when *f*_*Y*_ is non-Gaussian, then Eq (1) can be difficult to calculate. This can be addressed using Monte Carlo integration for the *E*-step [26], where replicates of ***X***_*k*_ = (*X*_1,*k*_, …, *X*_*n*,*k*_) for 1 ≤ *k* ≤ *p* are simulated and the *Q*-function is approximated by

$$Q\left(\beta |{\hat{\beta }}^{\left[t\right]}\right)\approx \frac{1}{B}\sum_{b=1}^{B}\;\text{log}\;\{f_{Y}\left(y|{\overset{ˇ}{x}}^{\left(b\right)};\beta \right)f_{W}\left(w|{\overset{ˇ}{x}}^{\left(b\right)}\right)f_{X}\left({\overset{ˇ}{x}}^{\left(b\right)}|{\hat{\beta }}^{\left[t\right]}\right)\}-h\left(\beta \right)$$

where ${\overset{ˇ}{x}}^{\left(b\right)}=\left[{\overset{ˇ}{x}}_{1}^{\left(b\right)}\cdots \;{\overset{ˇ}{x}}_{p}^{\left(b\right)}\right]$ and each ${\overset{ˇ}{x}}_{k}^{\left(b\right)}$ is sampled from the posterior distribution $f_{X}\left({\overset{ˇ}{x}}_{k}|{\hat{\beta }}^{\left[t\right]}\right)$ for *b* = 1, …, *B*, and *B* is the Monte Carlo sample size.

### Proposed implementation for measurement error modelling

While the MCEM algorithm, as originally proposed by [26], performs standard Monte Carlo integration sampling from the posterior distribution of the unobserved variable, in this paper, we will sample from the prior distribution and evaluate the *E*-step using importance sampling [27, 34]. Thus our *M*-step can be understood as fitting a model to “weighted” complete-data with importance weights, as in [35].

Specifically, we first sample replicate MC values for measurement error values ${\tilde{u}}^{\left(b\right)}=\left[{\tilde{u}}_{1}^{\left(b\right)}\cdots \;{\tilde{u}}_{p}^{\left(b\right)}\right]$ from the assumed measurement error distribution. We will sample from $N(0,\sigma_{u,k}^{2})$ for the *k*th corresponding error-contaminated covariate, as is usual in classical measurement error models, but in principle any known distribution could be used. We then construct *B* observed replicates of ***X*** denoted by ${\tilde{x}}^{\left(b\right)}=w-{\tilde{u}}^{\left(b\right)}$. Importance sampling can then be used to evaluate the *E*-step, as:

$$\begin{array}{c} Q\left(\beta |{\hat{\beta }}^{\left[t\right]}\right)\approx \sum_{b=1}^{B}q^{\left(b\right)}\left({\hat{\beta }}^{\left[t\right]}\right)\;\text{log}\;\{f_{Y}\left(y|{\tilde{x}}^{\left(b\right)};\beta \right)f_{W}\left(w|{\tilde{x}}^{\left(b\right)}\right)f_{X}\left({\tilde{x}}^{\left(b\right)}|{\hat{\beta }}^{\left[t\right]}\right)\}-h\left(\beta \right) \end{array}$$ (2)

with importance weights

$$q^{\left(b\right)}\left({\hat{\beta }}^{\left[t\right]}\right)=\frac{f_{Y}\left(y|{\tilde{x}}^{\left(b\right)};{\hat{\beta }}^{\left[t\right]}\right)f_{X}\left({\tilde{x}}^{\left(b\right)}|{\hat{\beta }}^{\left[t\right]}\right)}{\sum_{b=1}^{B}f_{Y}\left(y|{\tilde{x}}^{\left(b\right)};{\hat{\beta }}^{\left[t\right]}\right)f_{X}\left({\tilde{x}}^{\left(b\right)}|{\hat{\beta }}^{\left[t\right]}\right)}.$$

As is usual in EM-algorithms, the estimation procedure alternates between this *E*-step and a *M*-step that finds the maximiser of $Q(\beta |{\hat{\beta }}^{\left[t\right]})$ with respect to ***β***.

An important feature of our algorithm is that $Q(\beta |{\hat{\beta }}^{\left[t\right]})$ has the form of a weighted sum of complete data likelihoods, so fitting algorithms developed for error-free predictors can be applied, but to imputed and reweighted data. Note also that the *E*-step does not involve new data imputations, it just updates the weights for existing imputations. Thus our algorithm, summarised in Algorithm 1, can be understood as taking a fitting algorithm developed for error-free covariates, and applying it to imputed covariates in an iteratively reweighted fashion in order to account for measurement error.

**Algorithm 1** MCEM estimation algorithm with measurement error in covariates.

Consider an algorithm G(y;x;q) which estimates ***β*** by maximum likelihood or penalised likelihood from responses ***y***, predictors ***X*** and observation weights ***q***. We can extend this algorithm to estimate ***β*** by maximum (penalised) likelihood when there is measurement error in ***X*** that comes from a known distribution, such that we observe ***w*** only, as follows.

**Step 1**: Initialise model:

(a) Set initial values for ${\hat{\beta }}^{\left[t\right]}={\hat{\beta }}^{\left[0\right]}$ by fitting a naïve model, using the contaminated observed covariates w and no observation weights, via G(y;w;1).
(b) Simulate *B* replicate Monte Carlo values (denoted by ${\tilde{u}}^{\left(b\right)}$) for the measurement error, *b* = 1, …, *B*. Construct *B* replicates of ***X*** using ${\tilde{x}}^{\left(b\right)}=w-{\tilde{u}}^{\left(b\right)}$.
(c) Replicate ***y*** *B* times, ***y***^(*b*)^ = ***y*** for *b* = 1, …, *B*.

**Step 2**: Repeat until convergence of $\hat{\beta }$:

*E*-**step** Construct weights $q^{\left(b\right)}\left({\hat{\beta }}^{\left[t\right]}\right)\propto f_{Y}\left(y|\tilde{x};{\hat{\beta }}^{\left[t\right]}\right)f_{X}\left(\tilde{x}\right)$.
*M*-**step** Update model coefficients ${\hat{\beta }}^{\left[t+1\right]}$ by regressing the *B* replicates of ***y*** against the imputed covariates ${\tilde{x}}^{\left(b\right)}$ with observation weights $q^{\left(b\right)}\left({\hat{\beta }}^{\left[t\right]}\right)$, via $G(\left\{y^{\left(1\right)},\ldots ,y^{\left(B\right)}\right\};\left\{{\tilde{x}}^{\left(1\right)},\ldots ,{\tilde{x}}^{\left(B\right)}\right\};\left\{q^{\left(1\right)}\left({\hat{\beta }}^{\left[t\right]}\right),\ldots ,q^{\left(B\right)}\left({\hat{\beta }}^{\left[t\right]}\right)\right\})$.

### Algorithm properties

This MCEM algorithm has the key advantage of flexibility—in principle, it can be used to account for measurement error when fitting *any* regression model for which a maximum (penalised) likelihood fitting algorithm G(y;x;q) is already available in the case of error-free predictors. The reason we require G(y;x;q) to be a maximum likelihood algorithm is that our approach to measurement error modelling is itself motivated by maximum likelihood. Penalised likelihood algorithms, with a penalty on regression parameters ***β***, are also permissible since the penalty on the observed likelihood can be brought inside the integrand in Eq (2), leading to iteratively reweighted penalised likelihood.

Conditional on the Monte Carlo values ${\tilde{u}}^{\left(b\right)}$, Algorithm 1 is an EM algorithm. As such it inherits standard properties of an EM algorithm, in particular, that $\hat{\beta }$ converges to its local maximum, as in [32], under suitable regularity conditions, see also [36, 37]. Further, standard results for Monte Carlo integration [9] suggest that as the size of the Monte Carlo sample increases (*B* → ∞), our estimate of the *Q* function Eq (2) converges to the true *Q* function of Eq (1), whose maximiser is the maximum likelihood estimator. Thus, we have the following result.

**Theorem 1**. *Let* $\hat{\beta }$ *be the MCEM algorithm estimator of* ***β***. *Under the regularly conditions given in* [32], *and further assuming that f*_***X***_ *is known*, $\hat{\beta }\overset{P}{\to }\beta$, *as* *B* → ∞ *and* *n* → ∞.

We investigated the robustness of $\hat{\beta }$ when the model assumption on *f*_***X***_ was violated in a simulation study, see sub-section Robustness for results and discussion.

The algorithm is simplified by using a fixed set of Monte Carlo estimates of measurement error, which are not updated from one iteration to the next, only their importance weights are. In effect, this means we use ${\tilde{x}}^{\left(b\right)}=w-{\tilde{u}}^{\left(b\right)}$ as proposal values where ${\tilde{u}}^{\left(b\right)}$ are sampled from some prior distribution *f*_*U*_ rather than trying to sample ${\tilde{x}}^{\left(b\right)}$ from the posterior distribution *f*_*X*∣*Y*_. An important advantage of using fixed proposal values ${\tilde{u}}^{\left(b\right)}$ is that it stabilises the estimation algorithm, by removing Monte Carlo variation across iterations, and simplifying the algorithm to an EM algorithm (conditional on ${\tilde{u}}^{\left(b\right)}$). It is more typical [26, 28] to implement an MCEM algorithm by sampling directly from the posterior *f*_*X*∣*Y*_, hence all importance weights take the values 1/*B*. Because the distribution *f*_*X*∣*Y*_ is a function of the parameters being estimated, such Monte Carlo values would have to be updated every iteration, after parameters were updated.

Importance sampling works best when the proposal distribution closely matches the posterior. This can be diagnosed using importance weights, by computing the effective sample size (ESS) [38]

$$\begin{array}{c} \text{ESS}=\frac{1}{\sum_{b=1}^{B}{\left\{q^{\left(b\right)}\left(\hat{\beta }\right)\right\}}^{2}}. \end{array}$$ (3)

Generally speaking, we would expect that sampling from the prior *f*_*U*_ would only be appropriate in situations where the data (***y***, ***w***) are not to be very informative about the measurement error *U*. Because there is only one observation of (***y***, ***w***) for each measurement error value *U*, this seems reasonable here. Our diagnostic checks support this, with importance sampling weights suggesting effective sample sizes of the order of half of *B* or larger when there are three or less contaminated covariates. This means that, in our applications, we would need *B* to be about twice as large as what would have been the case when sampling directly from the posterior, in order to achieve similar accuracy in our Monte Carlo integral approximations. This we consider to be a modest cost to pay for generality and algorithm stability. Note however that the effective sample size decays as the number of contaminated covariates increases (see Fig S1.1 of S1 Appendix) or when the measurement error variance increases (see Fig S1.2 of S1 Appendix), so we should use this approach with caution when either is large.

As with any maximum likelihood approach applied to structural-type measurement error modelling, valid inference requires correct specification of the distribution for measurement error ***U***, and of the form of distribution for the predictors ***X***. We will assume both ***U*** and ***X*** come from multivariate Gaussian distributions, with a known measurement error covariance matrix **Σ**_*u*_ where the *k*th diagonal element is $\sigma_{u,k}^{2}$ and the (*k*, *l*)th element for *k* ≠ *l* is 0, as is common *e.g*., see [1]. In principle, any specified distribution can be used, but if ***X*** and ***U*** were not normal, the marginal distribution of ***W*** might no longer have a closed form. While this would not be a problem for estimation via Algorithm 1, it would make it more difficult to use the observed ***W*** to diagnose distributional assumptions on predictors. We use simulation later to illustrate the sensitivity of this modelling approach to violations of distributional assumptions on ***X***.

### Standard errors

To estimate standard errors for $\hat{\beta }$ we cannot use the Fisher information from the final (converged) model fit, as this uses the complete augmented data and ignores uncertainty in ***X***. This can result in underestimation of the population variances for ***β***. We follow [39] by using their proposed observed information to obtain standard errors when using the MCEM Algorithm 1.

Recall that ***X***_*i*_ = (*X*_*i*,1_, …, *X*_*i,p*_) be a vector of length *p*. Denote the score function for ***β*** as $S\left(Y,X;\beta \right)=\sum_{i=1}^{n}s\left(Y_{i},X_{i};\beta \right)$ and $E_{X|Y}\left\{I\left(Y,X;\beta \right)\right\}=-\sum_{i=1}^{n}E_{X|Y}\left\{J\left(Y_{i},X_{i};\beta \right)\right\}$ as the expected Fisher information function for ***β*** where *s*(*Y*_*i*_, ***X***_*i*_; ***β***) and *J*(*Y*_*i*_, ***X***_*i*_; ***β***) are the gradient and Jacobian functions, respectively. Recall that our final MCEM model fit uses each generated ${\tilde{x}}_{k}^{\left(b\right)}$ such that we have *B* weighted gradient functions and *B* weighted Jacobian functions. Following [39], the observed information for ***β*** is

$$\begin{array}{cc} I_{\hat{\beta }}\;\; & \equiv \;\;I_{W}\\ & =I_{X}-I_{X|W}\\ & =\underset{X|Y}{E}\left\{I\left(Y,X;\beta \right)\right\}-\underset{X|Y}{E}\left\{S\left(Y,X;\beta \right)S^{T}\left(Y,X;\beta \right)\right\}+S^{*}\left(\beta \right)S^{*T}\left(\beta \right), \end{array}$$ (4)

where $S^{*}\left(\beta \right)=E_{X|Y}\left\{S\left(Y,X;\beta \right)\right\}$. We replace expectations using the weighted average of *B* Jacobian functions and score functions, and substitute our estimates for ***β***. Also, note that $S^{*}\left(\beta \right)S^{*T}\left(\beta \right)=\sum_{i=1}^{n}E_{X|Y}\left\{s\left(Y_{i},X_{i};\beta \right)\right\}\cdot E_{X|Y}{\left\{s\left(Y_{i},X_{i};\beta \right)\right\}}^{T}$. From Eq (4), this yields:

$$\begin{array}{ccc} I_{W} & = & -\sum_{i=1}^{n}\underset{X|Y}{E}\left\{J\left(Y_{i},X_{i};\beta \right)\right\}-\sum_{i=1}^{n}\underset{X|Y}{E}\left\{s\left(Y_{i},X_{i};\beta \right)s^{T}\left(Y_{i},X_{i};\beta \right)\right\}+S^{*}\left(\beta \right)S^{*T}\left(\beta \right)\\ & \approx & -\sum_{i=1}^{n}\sum_{b=1}^{B}q_{i}^{\left(b\right)}\left(\hat{\beta }\right)\cdot J\left(y_{i},{\tilde{x}}_{i}^{\left(b\right)};\hat{\beta }\right)-\sum_{i=1}^{n}\sum_{b=1}^{B}q_{i}^{\left(b\right)}\left(\hat{\beta }\right)\cdot s\left(y_{i},{\tilde{x}}_{i}^{\left(b\right)};\hat{\beta }\right)\cdot s^{T}\left(y_{i},{\tilde{x}}_{i}^{\left(b\right)};\hat{\beta }\right)\\ & & \;+\;\sum_{i=1}^{n}[\{\sum_{b=1}^{B}q_{i}^{\left(b\right)}\left(\hat{\beta }\right)\cdot s\left(y_{i},{\tilde{x}}_{i}^{\left(b\right)};\hat{\beta }\right)\}\cdot {\left\{\sum_{b=1}^{B}q_{i}^{\left(b\right)}\left(\hat{\beta }\right)\cdot s\left(y_{i},{\tilde{x}}_{i}^{\left(b\right)};\hat{\beta }\right)\right\}}^{T}] \end{array}$$ (5)

where ${\tilde{x}}_{i}^{\left(b\right)}=\left({\tilde{x}}_{i,1}^{\left(b\right)},\ldots ,{\tilde{x}}_{i,p}^{\left(b\right)}\right)$ is a vector of length *p*. We then take $\hat{\text{Var}}\left(\hat{\beta }\right)\approx I_{W}^{-1}$ to obtain the standard errors for $\hat{\beta }$.

The calculation of *s*() and *J*() depends on the specified likelihood function of *Y*. If *Y* belongs to the exponential family, then both the gradient and Jacobian functions are easily obtainable. We used the sandwich R-package [40] to extract estimating functions for GLMs and GAMs in the Results section to evaluate Eq (4). We used a QR-decomposition on $I_{W}$ to speed up computation and match the same outputs when fitting lm() and glm().

**Remark**: If Monte Carlo error is too large, the estimate of $I_{W}^{-1}$ can be unreliable and even result in non-positive definitive matrices. A solution to this is to increase *B*. In our simulation studies and examples, we found that using *B* ≥ 50 gave stable estimates and standard errors for ***β***. As *B* be increases however, the computational cost also increases linearly with *B*.

## Results

### Simulation studies

We conducted several simulations studies to investigate the performance of MCEM under various settings, in particular: different distributions for the response variable, non-linearity between the response and error contaminated covariates, and different distributions for the true covariate. In all simulation studies below, we generated 200 data sets, and to obtain standard errors we used Eq (4) when using MCEM.

#### Bias, efficiency and confidence interval coverage

We first examined the relative bias, efficiency and coverage for regression coefficients with error contaminated covariates. We generated binary response data where the underlying true model was a quadratic (logistic) GLM conditional on one (true) covariate $X_{i}∼N\left(0,1\right)$ using a sample size of *n* = 800. The true parameter values were set to ***β*** = (0.5, 1, −0.3). We then added measurement error from $U_{i}∼N\left(0,\sigma_{u}^{2}\right)$ to the covariate to give the error-contaminated covariate *W*_*i*_. We compared MCEM results with the true GLM (*i.e*., a model using the true/error-free covariate *X*_*i*_), a naïve GLM (*i.e*., a model using *W*_*i*_) and SIMEX which we use as our benchmark model. We chose to compare SIMEX with MCEM in this simulation study, since SIMEX is able to flexibly fit logistic quadratic GLMs via the R-package simex [15].

**Fig 1:**
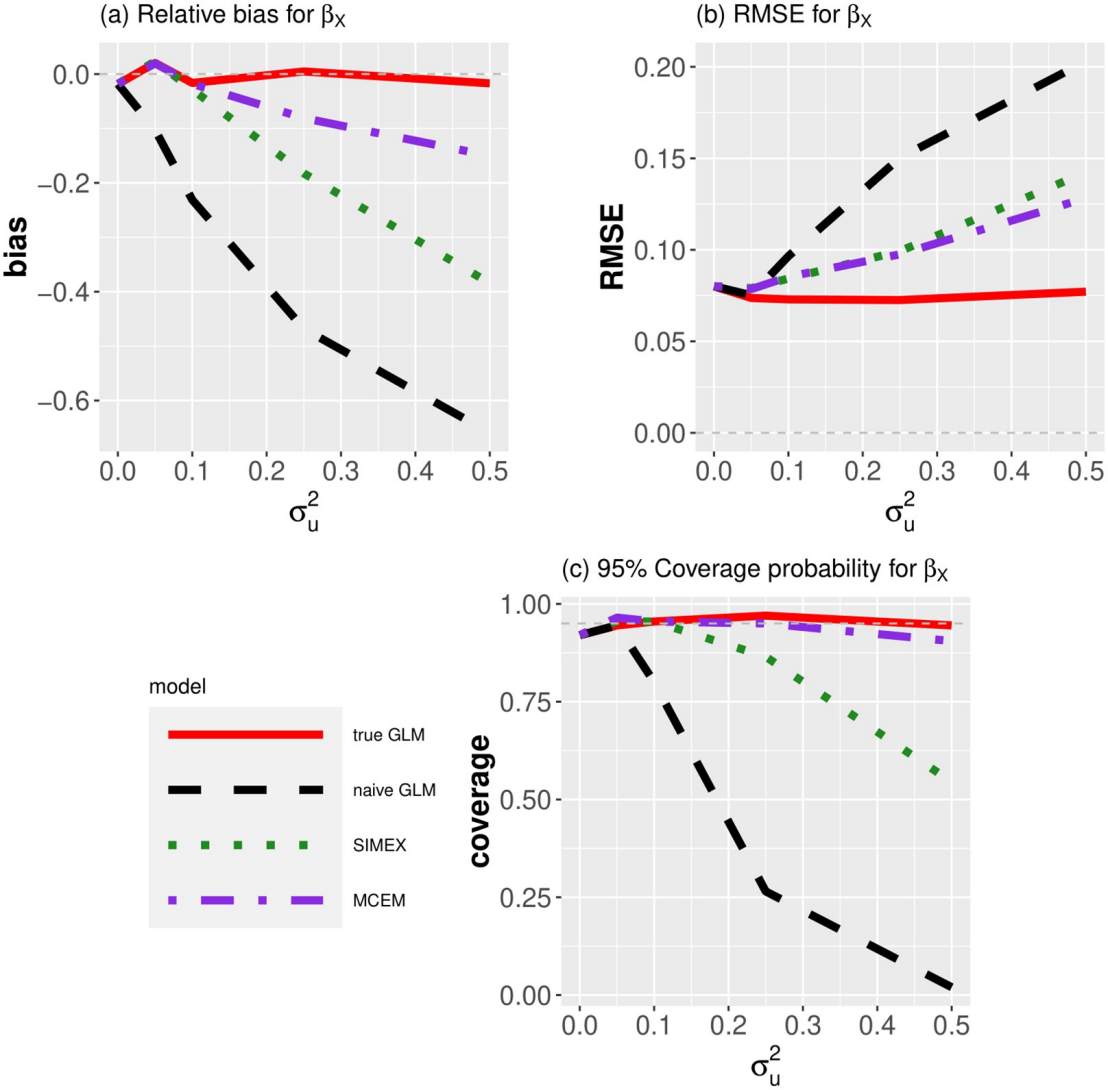
(a) Relative bias (b) RMSE and (c) 95% coverage probabilities for *β*_*X*_ when fitting quadratic (logistic) GLMs to binary data with one error-contaminated covariate across increasing measurement error values $\sigma_{u}^{2}$. We compare results when fitting a GLM using the true GLM (*i.e*., used the true covariate *X*_*i*_), the naïve GLM (*i.e*., used the error-contaminated covariate *W*_*i*_), SIMEX and MCEM. Note from (a) that MCEM has less relative bias than SIMEX and a naïve GLM, especially when the measurement error is large, and note from (c) that coverage probability of MCEM is close to nominal levels.

In Fig 1 we plotted the relative bias, RMSE and 95% nominal coverage probabilities for the quadratic effect term (*β*_*X*_ = −0.3) across increasing measurement error values $\sigma_{u}^{2}$. As expected, the naïve GLM resulted in large bias, RMSE and poor coverage, whereas the proposed MCEM model gave very little bias and excellent coverage (*i.e*., at the 95% nominal level). SIMEX still had an appreciable bias, hence poor coverage probability when the magnitude of measurement error was not small. Interestingly, the RMSE was similar for SIMEX and MCEM across all $\sigma_{u}^{2}$, indicating that the increased bias correction in MCEM also increased the variance.

#### Prediction

**Fig 2:**
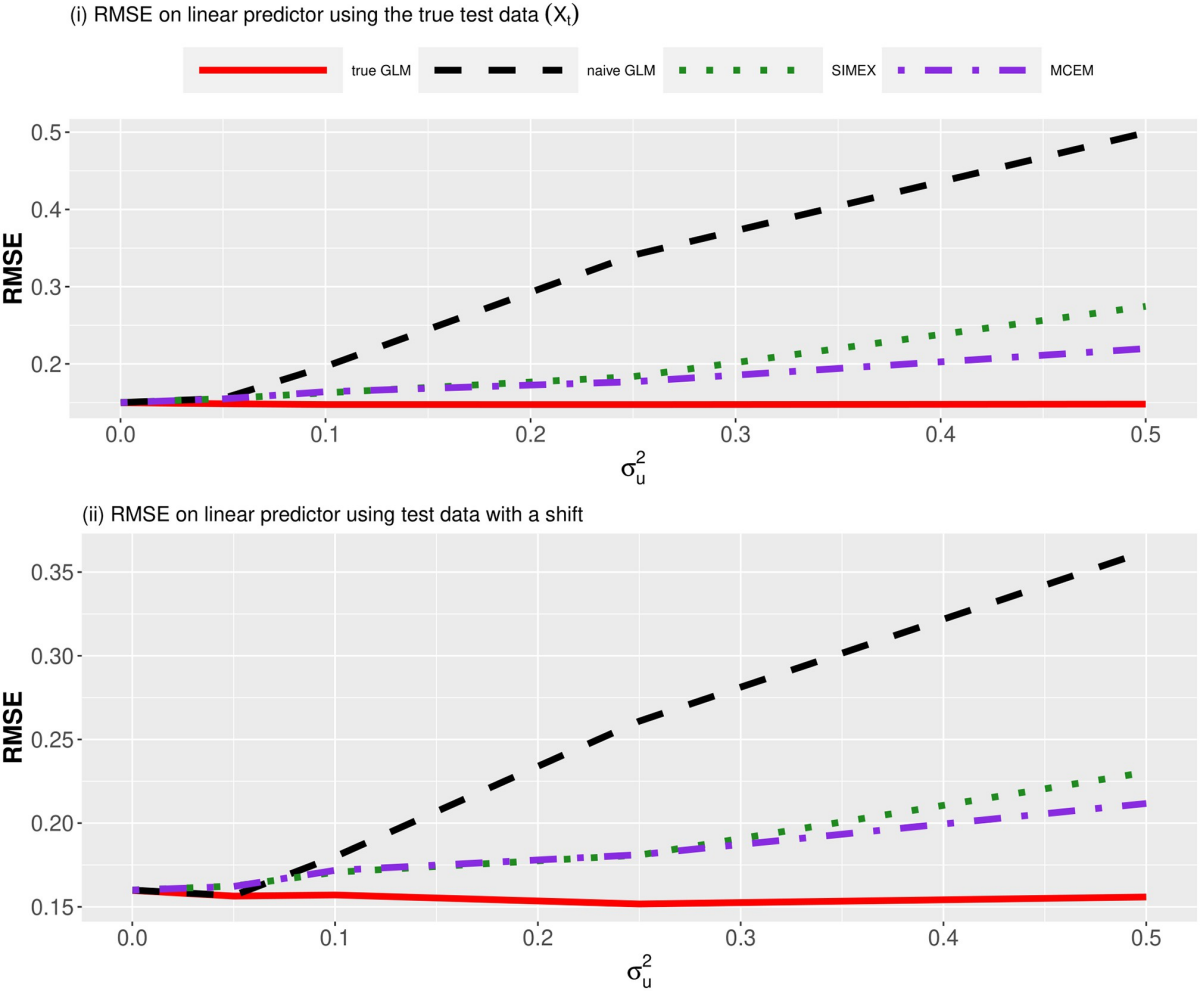
RMSE on the test data when fitting (logistic) quadratic models to binary data across increasing measurement error values $\sigma_{u}^{2}$. We considered two cases: (i) independent test data and (ii) independent test data with an increased shift of 0.5 units in *X*_*i*_. We fitted the same models as in Simulation 1. For both cases, we see that both MCEM and SIMEX give better predictions compared with the naïve GLM.

Next, we examined the predictive performance for MCEM on independent test data. First, we used the same (logistic regression) simulation set up and fitted the same models as in the previous section but now examined the RMSE on the linear predictor—*i.e*., we used RMSE (${\hat{\eta }}_{i})=\sqrt{\sum_{i=1}^{n^{*}}{\left(\eta_{i}-{\hat{\eta }}_{i}\right)}^{2}/n^{*}}$ where $\eta_{i}=X_{i}^{*}\beta$ and ${\hat{\eta }}_{i}=X_{i}^{*}\hat{\beta }$ denote the linear predictors, $X_{i}^{*}$ denotes the test covariate data and n* is the sample size of the test data. We investigated two cases: (i) independent test data, and (ii) independent test data with values of the covariate shifted upwards by 0.5 units, compared to values in training data. We considered a shift in *X*_*i*_ in test data to mimic the climate change scenario of Example 3 in the Results Section, where we expect that predictive performance of naïve methods will falter due to bias in the estimation of regression coefficients. The training sample was of size *n* = 800, and both cases for the test data were of size n* = 200. In Fig 2 we plotted the results across increasing measurement error values $\sigma_{u}^{2}$. For both cases, we see that both MCEM and SIMEX give much better predictions compared with the naïve GLM, with MCEM performing appreciably better than SIMEX under large levels of measurement error.

We also investigated the predictive performances of the MCEM approach for cases where there is a smooth non-linear relationship between a count response variable and the linear predictor—this mimics our examples given in the Results section. The smooth function between the response and predictor was set as *η*_*i*_ = cos(2*X*_*i*_ + 1/4). For MCEM, we model the non-linear relationship with splines using a GAM (via the mgcv package). We used default settings for the number of knots and spline basis. We fitted the true GAM, a naïve GAM and MCEM. In Fig S1.3 of S1 Appendix we plotted the RMSE on the test data across increasing measurement error values $\sigma_{u}^{2}$. Here we see that the naïve GAM performs poorly, this is because the smooth function is inadequately estimated by the naïve GAM on the training data, and is further compounded on the test data. MCEM lost considerable performance due to contamination of *X*_*i*_ for large $\sigma_{u}^{2}$, however its predictive performance was much better than the naïve GAM. We also ran a similar simulation for binary data and obtained similar results (not reported here).

#### Robustness

Finally, we examined the robustness of the MCEM approach. Recall that MCEM assumes a fixed prior distribution for *X*_*i*_. Algorithm 1 requires a distribution for *X*_*i*_ to be chosen in order to compute importance sampling weights q, although it does not impose any constraints on the type of distribution that is specified. In each of our examples, we assumed the true covariate was drawn from a normal distribution. We therefore investigated MCEM model performance under model mis-specification for *f*_*X*_(*x*_*i*_) and examined model robustness under these violations. Again, we considered the same simulation setup and fitted the same models as in the first simulation study but now generated: (i) $X_{i}∼\left(\chi_{3}^{2}-3\right)/\sqrt{6}$ and (ii) *X*_*i*_∣*κ* ∼ skewed normal distribution [41], the pdf for the skewed normal distribution is given by $f\left(x|\kappa \right)=2/\left(\kappa +\frac{1}{\kappa }\right)\{\phi (x/\kappa )I_{\left[0,\infty \right)}\left(x\right)+\phi \left(x\kappa \right)I_{\left(-\infty ,0\right)}\left(x\right)\}$ where *φ*(⋅) is the standard normal distribution. We set the skewness parameter *κ* to 3. We expected MCEM to do poorly due to the incorrect normality assumption on *X*_*i*_. Note that SIMEX makes no distributional assumptions on *X*_*i*_, so we expected that it may be less affected.

In Figs S1.4 and S1.5 of S1 Appendix, we once again plotted the relative bias, RMSE and 95% nominal coverage probabilities for the quadratic effect term across increasing measurement error values $\sigma_{u}^{2}$. We see that MCEM performed poorly in terms of both relative bias and RMSE (compared with the first simulation study) which is not surprising since the normality assumption is violated. However, the results were still usually better than SIMEX, which was surprising, for example the 95% coverage probabilities were reasonable for small $\sigma_{u}^{2}$ and case (i) although the performance did worsen as $\sigma_{u}^{2}$ increased. Thus MCEM seems to have a modest level of robustness to violations of distributional assumptions, although it can perform poorly in more extreme cases. Thus care should be taken to ensure distributional assumptions are reasonable. See also [42], who discuss goodness-of-fit testing of the error distribution in linear measurement error models.

### Applications

We present several real-data examples that demonstrate the utility of MCEM where maximum likelihood estimation is commonly used. In each example, a covariate (or covariates) is contaminated with measurement error. The first two illustratory examples enable a comparison of our approach to some well-known alternative approaches. The final two examples use more complex underlying models of interest to demonstrate the range of model types for our MCEM algorithm. Further details on all four examples are given in a refitME package vignette.

#### Example 1: Generalized linear models

We begin with a well-known example that uses data collected on male patients with coronary heart disease. The coronary heart disease data (a.k.a. the Farringham Heart Study) was analysed on page 112 of [1]. The response variable is binary (indicator of first evidence of CHD status) and there are four covariates: age, systolic blood pressure (SBP), smoking indicator and serum cholesterol level. The SBP covariate is known to be contaminated with measurement error where [1] estimated the measurement error variance to be $\sigma_{u}^{2}=0.00630$. The sample variance for SBP was $\sigma_{w}^{2}=0.0452$ which gave a reliability ratio of 86.1%. Throughout the paper we define the reliability ratio as $100\times (1-\sigma_{u}^{2}/\sigma_{w}^{2}$).

The model of interest here was a binomial GLM with a logit link function, including all four covariates, and measurement error on the SBP covariate. Table 1 reports results when using MCEM (as in Algorithm 1), a naïve GLM, or SIMEX, as in [1]. The number of simulations in SIMEX and Monte Carlo values were both set to *B* = 100 so that we could compare model fitting computational times.

**Table 1:** Model coefficient estimates (standard errors in parenthesis) for each model fitted to the Farringham Heart Study.

model	constant	SBP	chol. level	age	smoke
naïve GLM	-14.951 (1.900)	1.707 (0.418)	0.008 (0.002)	0.055 (0.012)	0.592 (0.250)
SIMEX	-15.919 (2.043)	1.947 (0.456)	0.008 (0.002)	0.053 (0.012)	0.598 (0.251)
MCEM	-16.059 (2.185)	1.955 (0.487)	0.008 (0.002)	0.056 (0.012)	0.594 (0.250)

Here, SBP = systolic blood pressure, chol. level = cholesterol level, age = age of patient and smoke = whether the patient is a smoker. The measurement error variance for SBP was estimated to be ${\hat{\sigma }}_{u}^{2}=0.0063$. Notice the difference between the GLM naïve and SIMEX/MCEM, and the similarity between SIMEX and MCEM for the SBP covariate coefficient estimate.

Even though the measurement error variance was relatively small, the difference between the GLM naïve and SIMEX/MCEM estimates for the SBP covariate was clearly evident. As expected, both measurement error models SIMEX and MCEM gave similar estimates and standard error for the SBP covariate. In terms of computational cost, MCEM was slightly faster for these data, as SIMEX required 9.559 secs and MCEM 4.507 secs (2.4 GHz 8-Core Intel Core i9, 16 GB 2667 MHz DDR4).

#### Example 2: Generalized additive models

Generalized additive models (GAMs) are commonly applied to data where the relationship between the response and linear predictor (or covariates) is non-linear. As discussed earlier, several methods have been developed to fit GAMs with measurement error in covariates, but with a focus on Gaussian responses. To fit these models, we can utilize Algorithm 1, since GAMs are typically fitted via penalised likelihood [17].

We used a real data example consisting of air pollution measures which were recorded in Milan, Italy from 1980 to 1989. These data were previously analysed in [22, 43], and are freely available from the SemiPar R-package [44]. The response variable of interest consists of daily mortality counts of individuals. Daily total suspended particles (TSP) measurements (on a log scale) were also collected and used as a covariate. Daily total suspended particles (TSP) measurements (on a log scale) were also collected and used as a covariate. Three additional covariates (sequential day number, average temperature and average relative humidity) were also used but only TSP was known to be measured with error [22]. All four covariates were assumed to have a non-linear relationship with the response.

Given the lack of validation data, the measurement error variance ($\sigma_{u}^{2}$) for the TSP covariate is unknown. To show that the TSP covariate was sensitive to measurement error, we follow [22] to conduct a sensitivity analysis by assuming different values for $\sigma_{u}^{2}$. [22] took a log-transformation on the response counts and assumed a Gaussian response with an additive structure using all four covariates. Here we fitted a MCEM Poisson GAM (with a log-link function) using all four covariates. To compare MCEM results with models that do not account for measurement error and the additive models of [22], we used the logarithm of TSP covariate with a low-to-moderate reliability ratio of 70% (*i.e*., the known measurement error variance was set to $\sigma_{u}^{2}=0.0915$). We also checked for over-dispersion for each model by inspecting residuals plots but found no apparent patterns.

**Fig 3:**
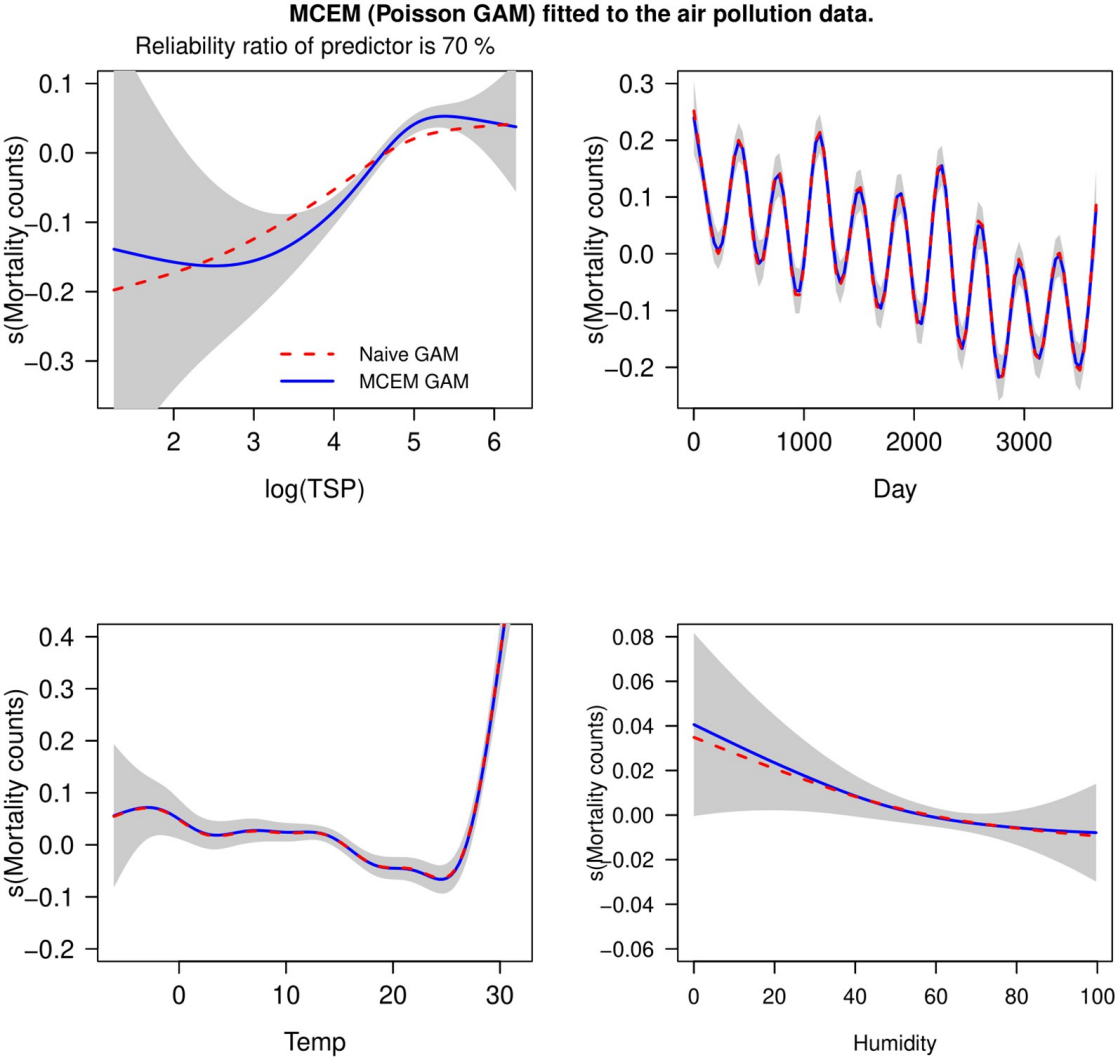
Poisson GAMs using MCEM (solid line) and a naïve model (dashed line) fitted to the air pollution data recorded in Milan, Italy 1980–1989. We also give 95% confidence bands. The response variable is total mortality counts. We plot the additive terms for (a) total suspended particles (log(TSP)), (b) day, (c) temperature, and (d) humidity. The log(TSP) covariate is assumed to have a reliability ratio of 70%.

In Fig 3, we plotted the additive terms for each covariate when fitting Poisson GAMs using MCEM and a naïve model. The estimated curves for each error-free additive term were very similar to naïve model fits and those given in Fig 2 of [22], see plots (b), (c) and (d). However the estimated MCEM curve for daily mortality counts against (log) TSP covariate was more non-linear (rather than strictly increasing) in comparison to the naïve model and the additive models of [22]. The refitME R-package (see the Software: The refitME function section) has been written so that plots of the subsequent gam object will use pointwise confidence bands that correct for measurement error, via the [39] method.

#### Example 3: Point process models

In this more complicated example we are interested in predicting species presence as a function of environmental covariates, and studying the potential impacts on species distribution of climate change. The main challenge here is that the species data are point event data, thus we wish to fit a point process regression model, for which measurement error software has not previously been available.

The data of interest are the spatial locations **s**_*P*_ of *m* = 89 reported sightings of the eucalypt species *Corymbia eximia* in the Greater Blue Mountains World Heritage Area and surrounding areas (denoted A), of size |A|≃86,000 square kilometres, about 100 kilometres west of Sydney, Australia. The world heritage area was given its designation in part because of its diversity of eucalypt species, so it is of considerable interest to understand how such species respond to the environment and key drivers of environmental change, like increasing temperatures.

Environmental covariates used in our model were average minimum daily temperature and annual rainfall. Data were opportunistically collected, rather than being based on systematic surveys, and as such we also attempt to model and correct for observer bias, using distance from nearest major road to reflect how accessible different areas of the study region was, as in [21, 45]. The *Corymbia eximia* data have been analysed previously in [20], but here we account for measurement in covariates and predict intensity of *Corymbia eximia* reportings as a function of these covariates. We will assume the intensity, or limiting number of presence reportings per unit area, has the form log λ(*s*) = **x**(*s*)′***β*** and estimate regression parameters ***β*** to maximise

$$l\left(\beta ;s_{P}\right)=\sum_{i=1}^{m}\;\text{log}\;\lambda (s_{i})-\int_{A}\lambda \left(s\right)ds$$

where *s*_*i*_ is the *i*th species reporting. The spatial integration was approximated using quadrature, with a regular 1-by-1 kilometre grid of *n* = 86, 227 quadrature points across A, enabling the following log-likelihood approximation:

$$\begin{array}{ccc} l(\beta ;s_{P}) & \approx & \sum_{i=1}^{m}\;\text{log}\;\lambda (s_{i})-\sum_{j=1}^{m+n}w_{j}\lambda \left(s_{j}\right)\\ & = & \sum_{j=1}^{m+n}w_{j}\{y_{j}\;\text{log}\;\lambda (s_{j})-\lambda \left(s_{j}\right)\} \end{array}$$

where *y*_*j*_ = 1/*w*_*j*_ for presence points (*j* = 1, …, *m*) and *y*_*j*_ = 0 otherwise. As quadrature weights, we use *ϵ* = 10^−6^ for presence points, and |A|/n for quadrature points [20]. The second line of the above is sometimes referred to as the Berman–Turner device [46], its benefit is that it expresses the Poisson process likelihood as a weighted sum of Poisson likelihoods, such that GLM software can be used for estimation, with weights on observations.

First, we fitted the naïve model—that is, we ignored measurement errors in the minimum temperature covariate **x**(*s*), and then applied Algorithm 1 assuming **x**(*s*) was subject to measurement error with reliability ratios of 93%, 87%, or 73%. These correspond to measurement error variance values of $\sigma_{u}^{2}=0.25,0.50$ and 1, respectively. To investigate future climate shifts and to project potential change in species distribution, we added 1.0° Celsius to the minimum temperature covariate, as this is the amount by which temperature would be expected to increase by 2050, assuming 0.20° Celsius per decade as estimated by [47].

**Fig 4:**
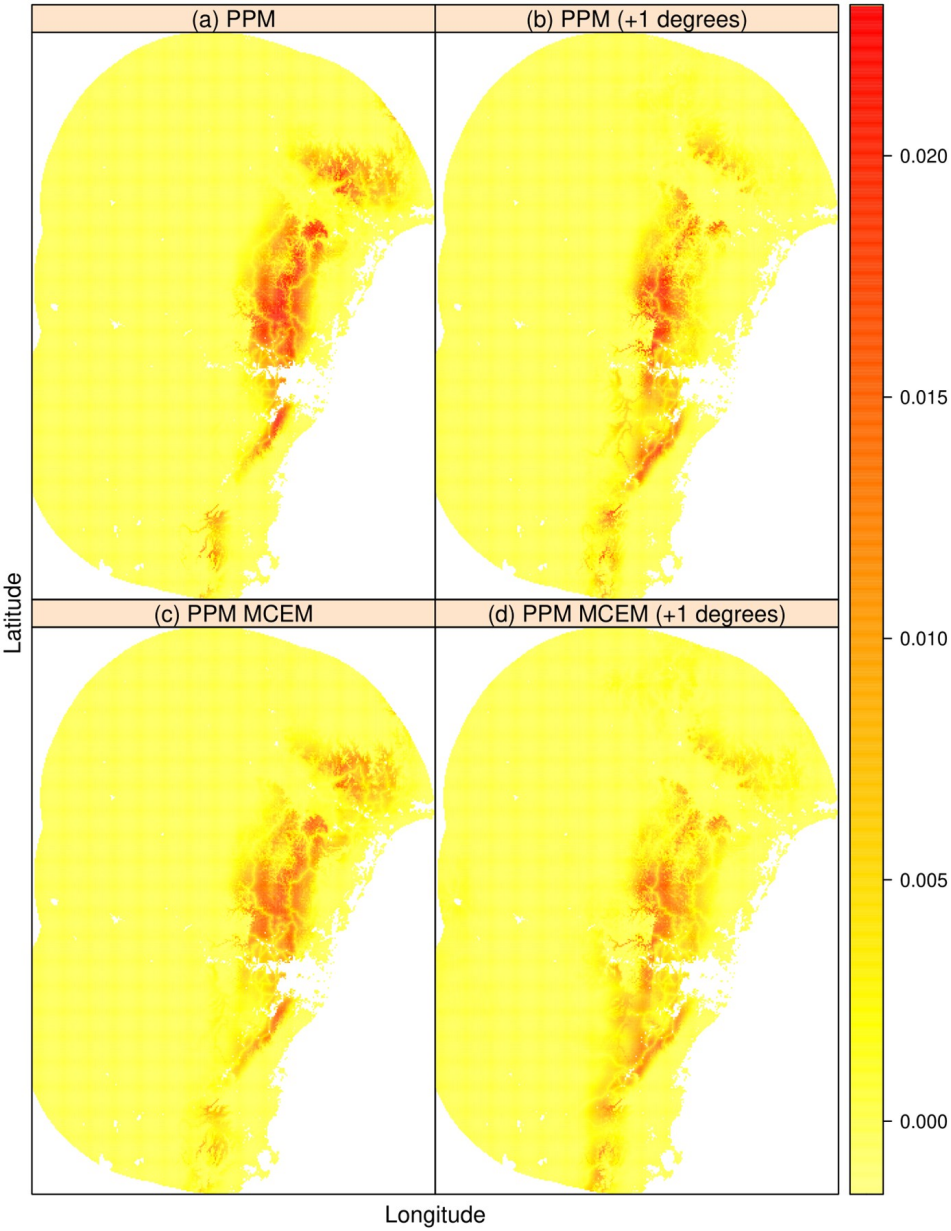
Predicted presences of *Corymbia eximia* using presence-only data. We assume the temperature covariate is subject to measurement error ($\sigma_{u}^{2}=1$ or with a reliability ratio of 73%). Models in the top row do not account for measurement error in the max temperature variable (reliability ratio of 100%) (a) PPM; (b) PPM with a 1.0 degree shift in max temperature; (c) PPM MCEM; and (d) PPM MCEM with a 1.0 degrees Celsius shift in max temperature.

In Fig 4 we plotted the predicted presences of *Corymbia eximia* using the covariates: minimum daily temperature, annual rainfall, and distance from nearest major road, where each covariate was modeled as a quadratic fit. The minimum daily temperature (assumed here to be error contaminated) had a reliability ratio of 73%. Models in the top row of Fig 4 do not account for measurement error in the minimum temperature variable (*i.e*., naïve PPM models). In Figs S1.6 and S1.7 of S1 Appendix, we give the same plots with reliability ratios of 93% and 87%, respectively. It is clear from both models that, if the species is to be found in areas with a similar climate to present-day, its distribution needs to move southward under the 1.0° Celsius warming scenario (Fig 4c and 4d). This conclusion is sensitive to measurement error in predictors, with the distance south that the species would need to move *increasing* as the reliability of minimum temperature measurements worsens, emphasising the importance of accurately estimating and accounting for measurement error in predictors.

#### Example 4: Capture–recapture models

Our final example illustrates how Algorithm 1 can be applied to a different class of model, the capture–recapture model, widely used in wildlife research.

A capture–recapture experiment consists of a sequence of capture occasions (labelled *t* = 1, …, *τ* where *τ* is the total number of capture occasions) upon which individuals in a population can be captured. The population size *N* is of unknown size and individuals are assumed to act independently of each other. The aim is to estimate the population size using information from the capture histories of individuals. Here, we assume the population is closed, that is, no births, deaths, emigration and immigration occur during the experiment.

Often, a two-step procedure is carried out where capture probabilities are modelled using observed covariates, denoted by *p*_*i*_ = *g*^−1^(*X*_*i*_***β***) with *g*^−1^(*u*) = exp(*u*)/{1 + exp(*u*)} being the logistic function. A conditional likelihood [48] can then be used to obtain estimates $\hat{\beta }$ and a Horvitz–Thompson estimator is employed to estimate the population size. The VGAM R-package [49] has in-built functions to fit a range of capture–recapture models (based on the conditional likelihood) and estimate population size via the Horvitz–Thompson estimator. See Web S1 Appendix for details and additional simulation studies.

In this study we use a well-known capture–recapture dataset collected on the yellow-bellied Prinia *Prinia flaviventris* in Hong Kong which has been analysed extensively in various studies over the last few decades—*e.g*., see [50, 51]. Bird wing lengths were also measured in the study and used as a covariate to model capture probabilities. However, these bird wing lengths are known to be subject to imprecise measurements, see [25]. Furthermore, several studies have identified a non-linear relationship between bird wing length and capture probability, see [52]. Although [25] corrected for measurement error in the wing length covariate, they mentioned that their methods *“were restricted to simple linear models and relaxation of this restriction may extend the applicability of the method but could require the development of more sophisticated methods”*.

We simultaneously address measurement error in covariates and non-linearity by using the MCEM algorithm described in the Materials and methods section. Specifically, we use a weighted conditional likelihood via the weights argument in VGAM, and non-parametrically model the capture probabilities using *B*-splines basis functions—this is also easily done in VGAM by using the s() function. There were *D* = 164 uniquely captured birds across *τ* = 17 weekly capture occasions. The sample mean for the bird wing length covariate was 45.230 mm, the sample variance was $\sigma_{w}^{2}=1.562$ mm and the estimated measurement error variance was ${\hat{\sigma }}_{u}^{2}=0.37$ mm, thus the reliability ratio for the bird wing length covariate was approximately 81%.

We fitted naïve conditional likelihood models (using vglm() and vgam() where the latter accounts for smoothing), the conditional score approach of [50] which accounts for measurement error but no smoothing, and the MCEM model which accounts for both. For each model we used the posbinomial() family provided in VGAM. We reported the AIC values (note that for the conditional score this was not available) and the population estimates along with their standard errors in Table 2. For the MCEM model, we used a weighted Horvitz–Thompson estimator (see Web Appendix S1 for details). Currently, the refitME package does not support all VGAM families, it can only fit the capture–recapture models described above.

**Table 2:** AIC and population size estimates (standard errors in parenthesis) for each model fitted to prinia bird capture–recapture data.

model	AIC	$\hat{N}$
naïve VGLM	1293.51	487.94 (85.20)
conditional score	–	528.99 (101.82)
naïve VGAM	1291.50	555.15 (119.03)
MCEM VGAM	1272.87	633.11 (172.35)

From Table 2, we see that the population size estimate was larger for the conditional score compared with the naïve VGLM which agrees with the results of [50]. Based on AIC, the naïve VGAM suggested a slightly better fit to the data *c.f*. the naïve VGLM, suggesting that some non-linearity is present. The population size for the naïve VGAM was also larger for the naïve VGAM. When accounting for both smoothness and measurement error, we see that MCEM yielded a much smaller AIC and had resulted in a larger population size.

### Software: The refitME function

We have written an R-package refitME to implement our algorithm. It is so-named because usage is analogous to the refit() function—takes a naïve model object, fitted to data without accounting for measurement error, and refit the model, but now assuming measurement error that follows a user-specified distribution. The code is written in a generic way so that it will add measurement error to any naïve model object that respond to a few generic R functions (family, model.frame, update and predict) and accepts the argument weights. It will additionally return standard errors, if the naïve object is supported by the sandwich package. The refitME function works by imputing values for ***X*** using the given measurement error model and applying Algorithm 1.

The refitME() function requires as input the fitted (naïve) model object and the variance of measurement error on covariates:


refitME(mod, sigma.sq.u, B = 50, epsilon = 1e-05, 
                                           silent = FALSE, ...)


The arguments are as follows:

- mod: any (S3 class) fitted object that responds to the generic functions family, model.frame, update and predict and accepts weighted observations via weights. The mod argument specifies the naïve fitted model. Make sure the first *p* input predictor variables in the naïve model are the selected error-contaminated variables (*i.e*., the first *p* predictors should correspond to the entries in *W*. The mod argument also allows a vlgm/vgam (S4 class) model objects when using the posbinomial family—this is a specific function developed for fitting closed population capture–recapture models, see Example 4: Capture–recapture model,
- sigma.sq.u: the known measurement error variance (*i.e*., , the **Σ**_*u*_). A scalar if there is only one error-contaminated variable, otherwise this must be stored as a vector or a matrix if the measurement error covariance matrix is known.
- B: the number of Monte Carlo replication values (default is set to 50).
- epsilon: convergence threshold (default is 0.00001).
- silent: if TRUE, the “convergence message” (which tells the user if the model has converged and reports the number of iterations required) is suppressed (default is set to FALSE).
- …: further arguments passed through to the function that was used to fit mod, that will be used in refitting. These need only be specified if making changes to the arguments as compared to the original call that produced mod.

The refitME() function returns an object of the same form as the original naïve fitted model object (mod) but where coefficient estimates, the covariance matrix, fitted values, the log-likelihood, and residuals have been computed to account for measurement error via MCEM. Standard errors (see the Standard errors Section) are included and returned, if mod is a class of object accepted by the sandwich package (such as glm, gam, survreg and many more). Also returned are the measurement error variance and the effective sample size (ESS) which diagnose how closely the proposal distribution matches the posterior, see Eq (3). The observed log-likelihood is returned rather than the *Q* function (2), obtained by subtracting off the entropy term [53]. Generic functions, like summary(), AIC(), anova(), *etc*. can then be applied to the fitted refitME model object, to make inferences from the model that account for user-specified measurement error in covariates.

Below, we document fitting the MCEM algorithm via the refitME R-package for GLMs and the coronary heart disease data (see Example 1: Generalized linear models).


R> # Load data and R-packages.
R> data(Framinghamdata)
R> sigma.sq.u <- 0.006295 # ME variance from Carroll et al.


The first stored variable w1 is the error contaminated variable used in the analysis.


R> glm_naiv <- glm(Y ~ w1 + z1 + z2 + z3, x = TRUE,
+                   family = binomial, data = Framinghamdata)
R> B <- 100  # No. Monte Carlo replication values.
R> glm_MCEM <- refitME(glm_naiv, sigma.sq.u, B)
R> summary(glm_MCEM)


## Discussion

In this study we developed a new unified algorithm for flexible regression-type measurement modelling via MCEM. Provided that: (1) the error-free (true) covariate follows its assumed distribution; and (2) the distribution of measurement error is known (including parameters, most critically, measurement error variance), then MCEM can be easily incorporated for any regression model. Algorithm 1 is similar to an MCEM algorithm used in covariance modelling of non-Gaussian data using Gaussian copulas [54]—that algorithm also samples from the prior, for similar reasons, and also has the advantage of flexibility, in being able to combine data from any parametric marginal model with any covariance modelling algorithm designed for Gaussian data.

While relatively little software is available that can be used as a basis for comparison with our method, for simple parametric models, SIMEX software can be used via the simex package. In this setting we found that MCEM had relatively little bias and relatively reasonable coverage probability (see Fig 1), and was also slightly faster when using the same number of SIMEX simulations and replicate Monte Carlo values (*B*). In Examples 2 to 4, we demonstrated the simplicity of fitting MCEM to more complicated model structures for which measurement error modelling techniques had not previously been developed—GAMs with count data, PPMs on presence-only data and zero-truncated count models for capture–recapture data.

Evidently, there are many more potential models to which our proposed MCEM algorithm could be applied to develop errors-in-variables extensions. One technique of particular note is penalised likelihood methods using penalties that introduce sparsity, including the LASSO and its generalisations [55], used for example for model selection when there are many covariates [2, 3, Section 6.3]. However, care should be taken when there are many covariates, as we do not expect our importance sampling algorithm to scale well as the number of contaminated covariates gets large, without using a more targeted importance sampler. The MCEM algorithm could also be extended to handle missing data in covariates, see [56], and when there is significant correlation with the dependent variable, see [29, 57] who used the MCEM algorithm on correlated multivariate data where the premise in their study was on the implementation of random effects. Some further investigation is still required to see how well the MCEM method performs when the covariates (or even the measurement errors) are correlated with each other and when using categorical covariates that are subject to misclassification.

Our algorithm is implemented in a generic way, so that measurement error can be added to any naïve model fit that responds to some generic functions (family, model.frame, predict and update) and accepts weighted observations via the weights argument. In future research, it would be particularly useful to develop a refitME implementation for mixed modelling compatible with nlme or lme4. Also of interest is developing compatibility with a broader range of family types such that refitME could handle ordered logistic or probit regression models using the polr() function via the MASS package, and survival analysis using the coxph() function via the survival package.

Applied statistics is becoming increasingly sophisticated, with innovations in model-fitting algorithms being developed all the time, but often without errors-in-variables functionality. Our MCEM measurement error modelling algorithm is readily generalisable, essentially acting as a wrapper function around any given algorithm, with contaminated covariates handled via iteratively reweighted refits to imputed data. As such this approach can be used going forward to construct measurement error modelling extensions of new regression techniques as they are developed, provided that they are fitted by maximum or penalised likelihood techniques. This offers the potential for measurement error modelling using most regression modelling tools available today, or even tomorrow!

## Supporting information

S1 Appendix Supporting information containing further capture–recapture details with simulations and web figures (Fig S1.1–S1.7). (PDF)
