# Supplementary material for: A general algorithm for error-in-variables regression modelling using Monte Carlo expectation maximization
Source: PLoS One. 2023 Apr 3;18(4):e0283798. doi: 10.1371/journal.pone.0283798 (PMC10069785; doi:10.1371/journal.pone.0283798)
Supplement: S1 Appendix — (PDF) [file pone.0283798.s001.pdf]

S1 Appendix: A General Algorithm for Error-in-variables Regression Modelling using Monte Carlo Expectation Maximization

Jakub Stoklosa*[1], Wen-Han Hwang[2], and David I. Warton[1]

[1]School of Mathematics and Statistics and Evolution & Ecology Research Centre, The University of New South Wales, Sydney, Australia

[2]Institute of Statistics, National Chung Hsing University, Taiwan

*Corresponding author: j.stoklosa@unsw.edu.au; School of Mathematics and Statistics and Evolution & Ecology Research Centre, The University of New South Wales, Sydney, 2052, NSW, Australia

The following supporting information contains further capture–recapture details with simulations and web figures (Fig S1.1–S1.9).

Further capture–recapture details and simulations

Notation and error-free capture–recapture modelling

Let N be the unknown population size of individuals within the area of interest. Label the individuals so that $i = 1, \ldots, D$ are the captured and $i = D+1, \ldots, N$ are the uncaptured individuals for τ capture occasions. Let $Y_i = \sum_{j=1}^{\tau} Y_{it}$ be the number of times the ith individual has been caught in the experiment where Y_{it} is the indicator that the ith individual has been caught on the tth occasion. Suppose there is no measurement error, and recall that a parametric form for capture probabilities is denoted by $p_i = g^{-1}(X_i\boldsymbol{\beta}^{\mathsf{T}})$ with $g^{-1}(u) = \exp(u)/\{1+\exp(u)\}$ being the logistic function. Let $q_i = 1 - p_i$ and $\pi_i = 1 - q_i^{\tau}$ be the probability individual i is captured at least once. Covariates for uncaptured individuals are clearly not known, thus we need to use a conditional likelihood (Huggins, 1989), written as:

$$\ell_C(\boldsymbol{\beta}) = \prod_{i=1}^{D} \left\{ \frac{p_i^{Y_i}(1-p_i)^{\tau - Y_i}}{\pi_i} \right\} \tag{1}$$

and is maximized w.r.t. β. To estimate the population size, once an estimator $\widehat{\beta}$ of β is available, the Horvitz–Thompson estimator $\widehat{N} = \sum_{i=1}^{D} 1/\widehat{\pi}_i$ may be used. Variance estimates for $\widehat{N}$ are computed through a Taylor's series approximation (Huggins, 1989).

Capture–recapture modelling using MCEM

Suppose now that X_i is measured with (additive) measurement error such that we use the error-contaminated covariates W_i. We use the MCEM procedure described in the Materials and methods section of the main text where $p_i = g^{-1}\{H(W_i)\}$ and $H()$ is an unknown smooth function. We now use a weighted conditional likelihood via the `weights` argument in `VGAM`, and non-parametrically model the capture probabilities using B-splines basis

functions – this is also easily done in VGAM by using the s() function. Due to the required weighting in the conditional likelihood, we also require a weighted Horvitz–Thompson estimator to estimate the population size $\widehat{N}_q = \sum_{b=1}^{B}\sum_{i=1}^{D} q_i^{(b)}(\widehat{\boldsymbol{\beta}})/\widehat{\pi}_i^{(b)}$ where $\widehat{\pi}_i^{(b)}$ and $q_i^{(b)}(\widehat{\boldsymbol{\beta}})$ are both obtained from the final iteration of the MCEM model fit. Note that $\widehat{N}_q$ is an unbiased estimator for N. To see this let $\mathcal{C}_i$ take the value 1 if the ith individual has been captured at least once and 0 otherwise. Note that $\mathrm{E}(\widehat{N}) = \mathrm{E}\{\sum_{i=1}^{D} 1/\widehat{\pi}_i\} = \mathrm{E}\{\sum_{i=1}^{N} \mathbf{I}(\mathcal{C}_i)/\pi_i\} = N$, since $\mathrm{E}\left\{\mathbf{I}(\mathcal{C}_i)\right\} = \pi_i$, this yields

$$\mathrm{E}(\widehat{N}_q) = \sum_{b=1}^{B}\sum_{i=i}^{N} \frac{q_i^{(b)}(\widehat{\boldsymbol{\beta}})\mathrm{E}[\mathbf{I}\{\mathcal{C}_i^{(b)}\}]}{\pi_i^{(b)}} = N$$

Variance estimates for $\widehat{N}_q$ can be easily obtained using Taylor's series approximation and Louis' formula as given in the Standard errors section of the main text. Below, we conducted a simulation study to check the validity of the proposed method and $\widehat{N}_q$.

Simulation study

We constructed a simulation study to examine how well the MCEM approach performed on closed population capture–recapture data. We also examined how well the above population size estimator $\widehat{N}_q$ performs. We fix the the true population size to $N = 200$, and set the number of capture occasions to $\tau = 7$. We generated the covariate X_i for $i = 1, \ldots, N$ from the standard normal distribution. Capture probabilities (p_i) were generated by setting $\boldsymbol{\beta}$ to a linear model where $\boldsymbol{\beta} = (-1, 1)$ which we called case (i) and a quadratic model where $\boldsymbol{\beta} = (0.5, 0, -0.3)$ called case (ii). For each i, the observed covariate was then generated as $W_i = X_i + U_i$, where the $U_i \sim \mathcal{N}(0, \sigma_u^2)$ distributed. The measurement error variance σ_u^2 was set at various levels. To compare our results, we fitted the naïve conditional likelihood (CL), a conditional score (CS) model, and the above MCEM approach. Further details on the CS can be found in Hwang, Huang and Wang (2007). For case (ii), we fitted a linear model for CS only since quadratic models are not available.

In Figs S1.8–S1.9 we plotted the relative bias, RMSE and 95% coverage probabilities for N for both cases (we omitted results for $\boldsymbol{\beta}$). Clearly, MCEM outperformed the naïve CL

approach in both cases, and gave comparable bias, RMSE and 95% coverage probabilities to both the true CL and CS for case (i). For case (ii), MCEM outperformed CS in terms of bias and 95% coverage probabilities, this was expected since a linear model was used for CS. The RMSE was however slightly larger.

References

Huggins, R. M. (1989). On the statistical analysis of capture experiments. *Biometrika* **76**, pp. 113–140.

Hwang, W. H., Huang, S. Y. H., and Wang, C. Y. (2007). Effects of measurement error and conditional score estimation in capture–recapture models. *Statistica Sinica* **17**, pp. 301–316.

Web Figures

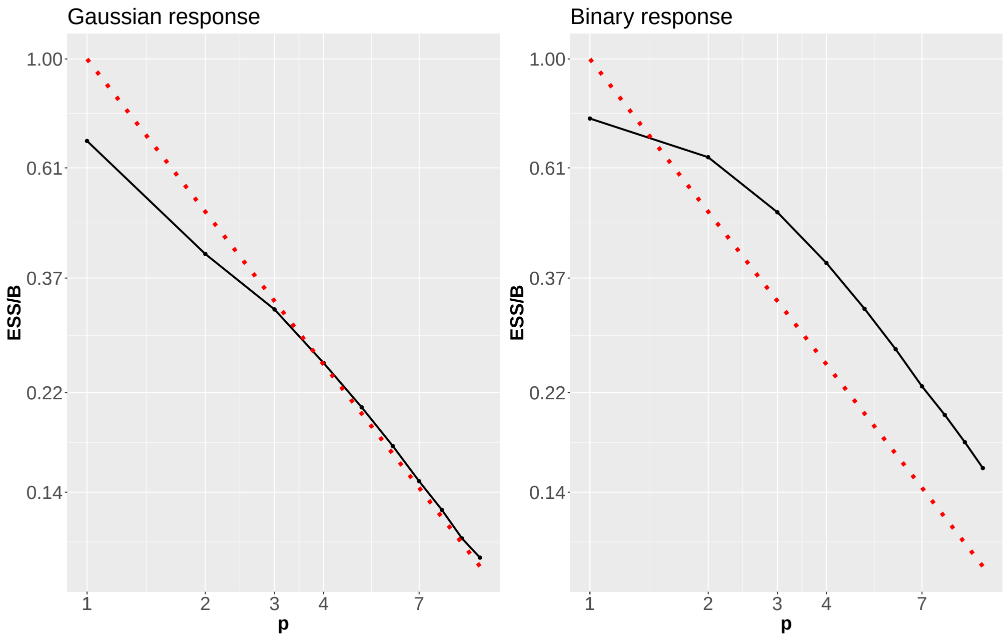

S1. 1: *Average effective sample size (ESS) divided by the number of Monte Carlo replications* B *against the number of error-contaminated covariates/dimension* (p) *on a log-log scale for Gaussian and binary response data (black thick lines). The red dotted line represents the dimension* p *against* $1/p$*, also on the log-log scale. Note the similarity between the two lines. The sample size was* $n = 10000$*, the number of Monte Carlo replications was* $B = 50$*, the true covariates were generated from the standard normal distribution with added Gaussian measurement errors* ($\sigma^2_{u,0} = 0, \sigma^2_{u,k} = 0.50$) *and parameter coefficients set to* $\boldsymbol{\beta} = (\beta_0, \beta_1, \ldots, \beta_k) = (0.5, 1, \ldots, 1)$ *for* $k = 1, \ldots, p = 10$.

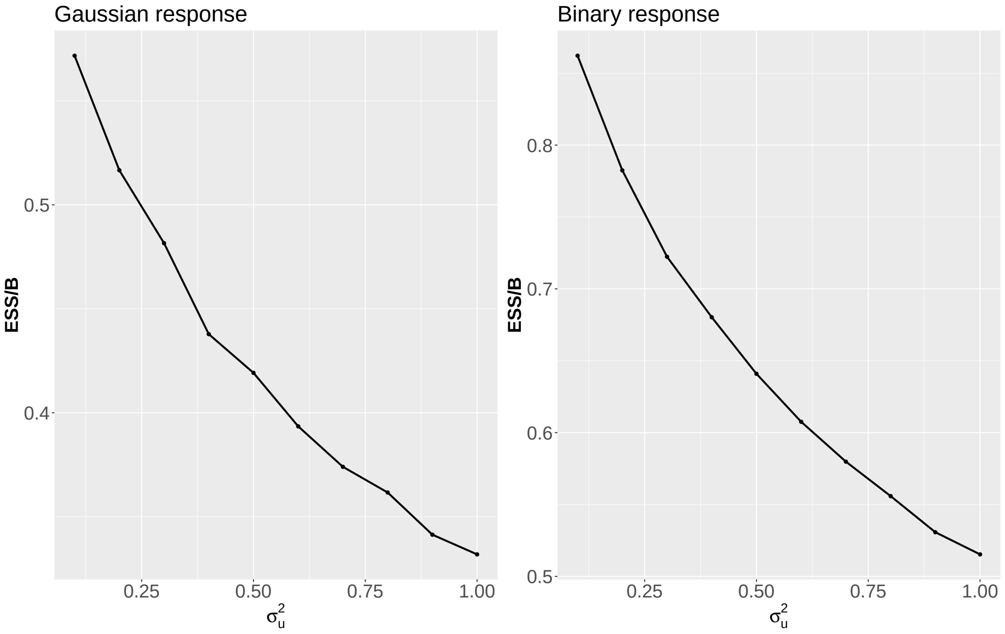

S1.2: *Average effective sample size (ESS) divided by the number of Monte Carlo replications* B *across increasing measurement error values* σ_u^2 *for Gaussian and binary response data. The sample size was* $n = 10000$*, the number of Monte Carlo replications was* $B = 50$*, the covariate dimension was* $p = 2$*, the true covariates were generated from the standard normal distribution with added Gaussian measurement errors with* $\sigma_{u,0}^2 = 0, \sigma_{u,1}^2 = \sigma_{u,2}^2 = 0.1, 0.2, \ldots 1$ *and* $\boldsymbol{\beta} = (\beta_0, \beta_1, \beta_2) = (0.5, 1, 1)$.

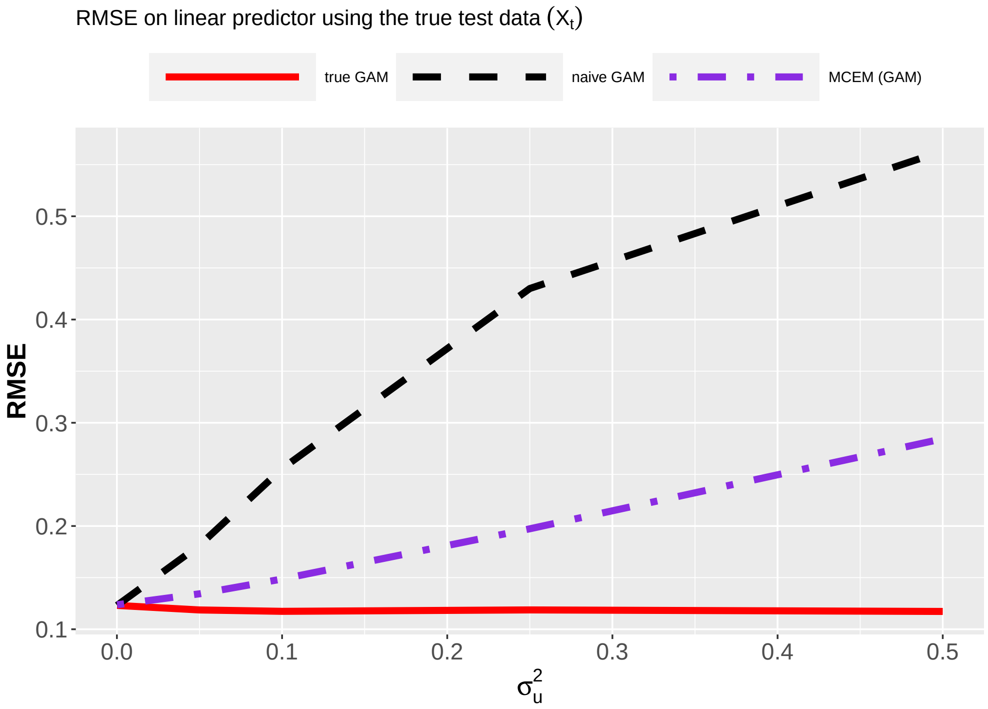

S1.3: *RMSE on the test data when fitting GAMs across increasing measurement error values* σ_u^2 *where relationship between the response and linear predictor is a non-linear smooth function. We compare results when fitting a GAM using the true GAM* (i.e., *uses the true covariate* X_i*), the naïve GAM* (i.e., *uses the error-contaminated covariate* W_i*) and MCEM.*

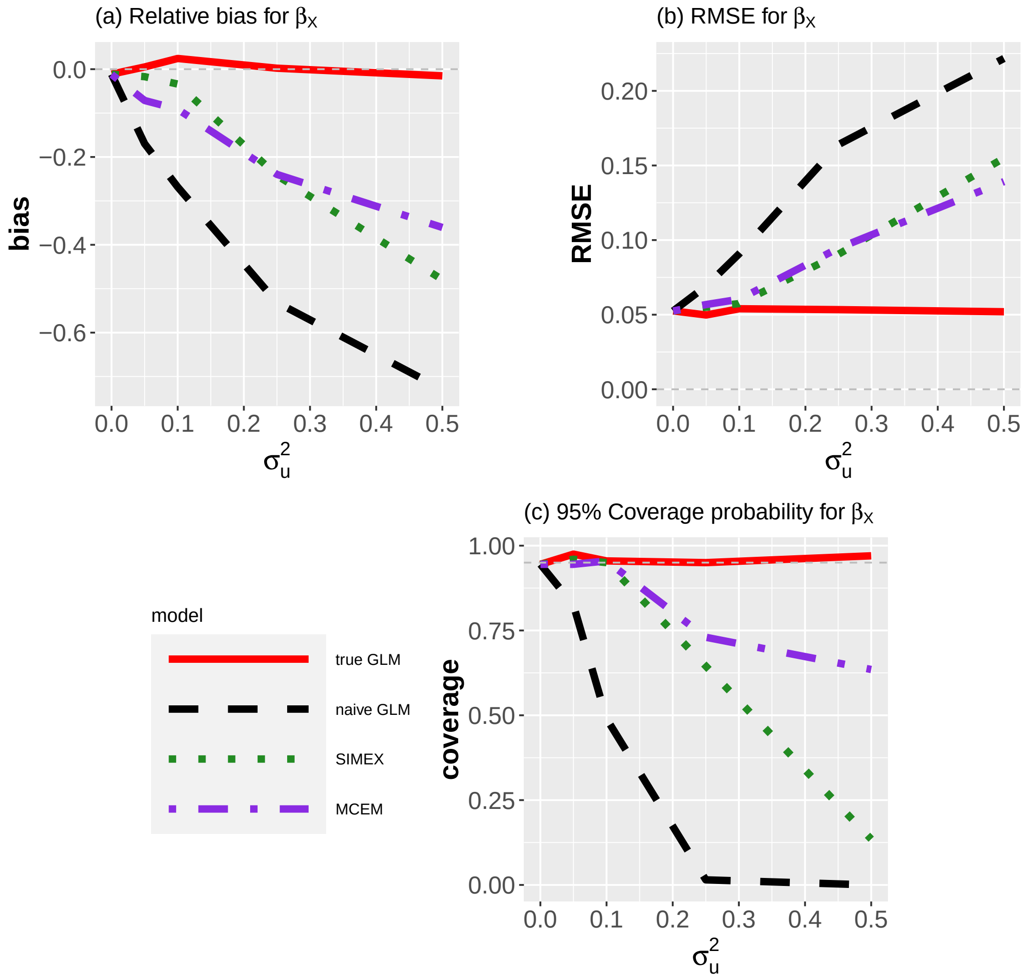

S1.4: *(a) Relative bias, (b) RMSE and (c) 95% coverage probabilities for* β_X*. Note that this is the same set up as in Section 3.1 but we now use* $X_i \sim (\chi_3^2 - 3)/\sqrt{6}$*.*

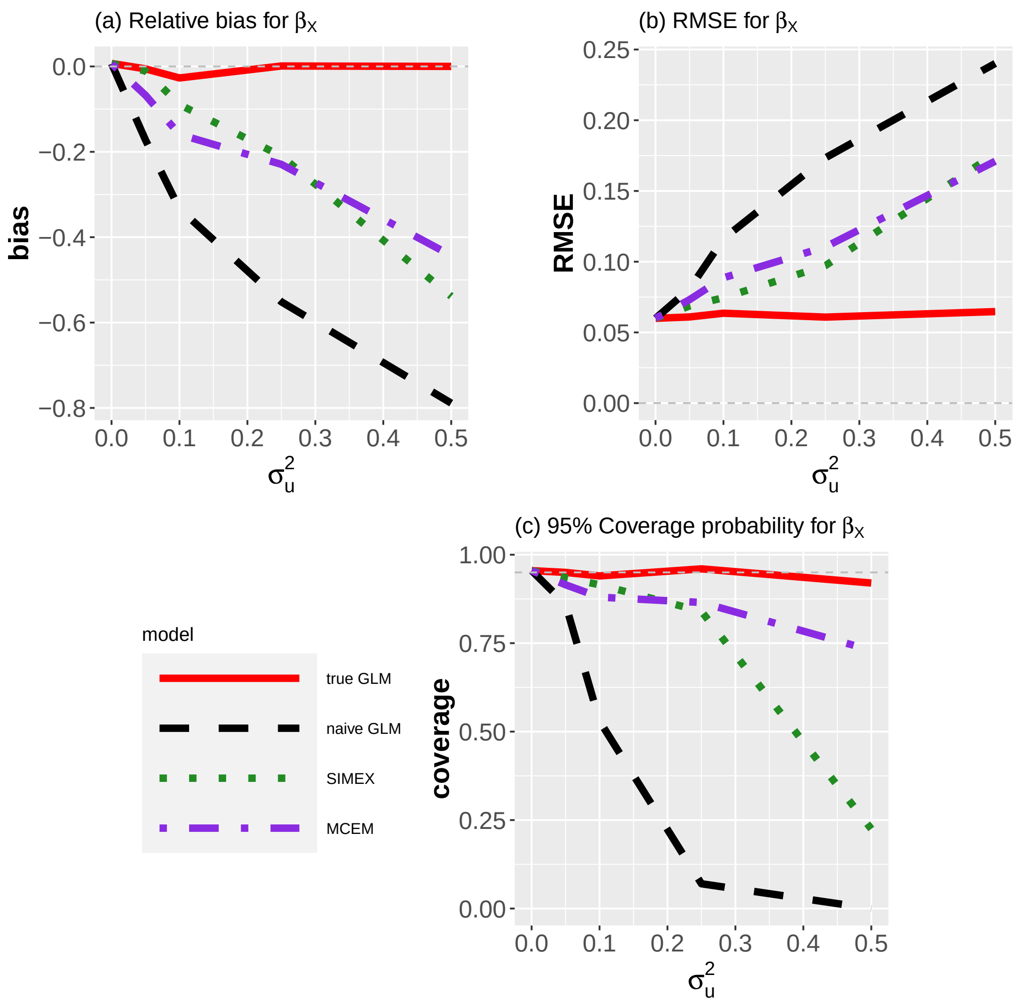

S1.5: *(a) Relative bias, (b) RMSE and (c) 95% coverage probabilities for β_X. Note that this is the same set up as in Section 3.1 but we now use a skewed normal distribution for X_i.*

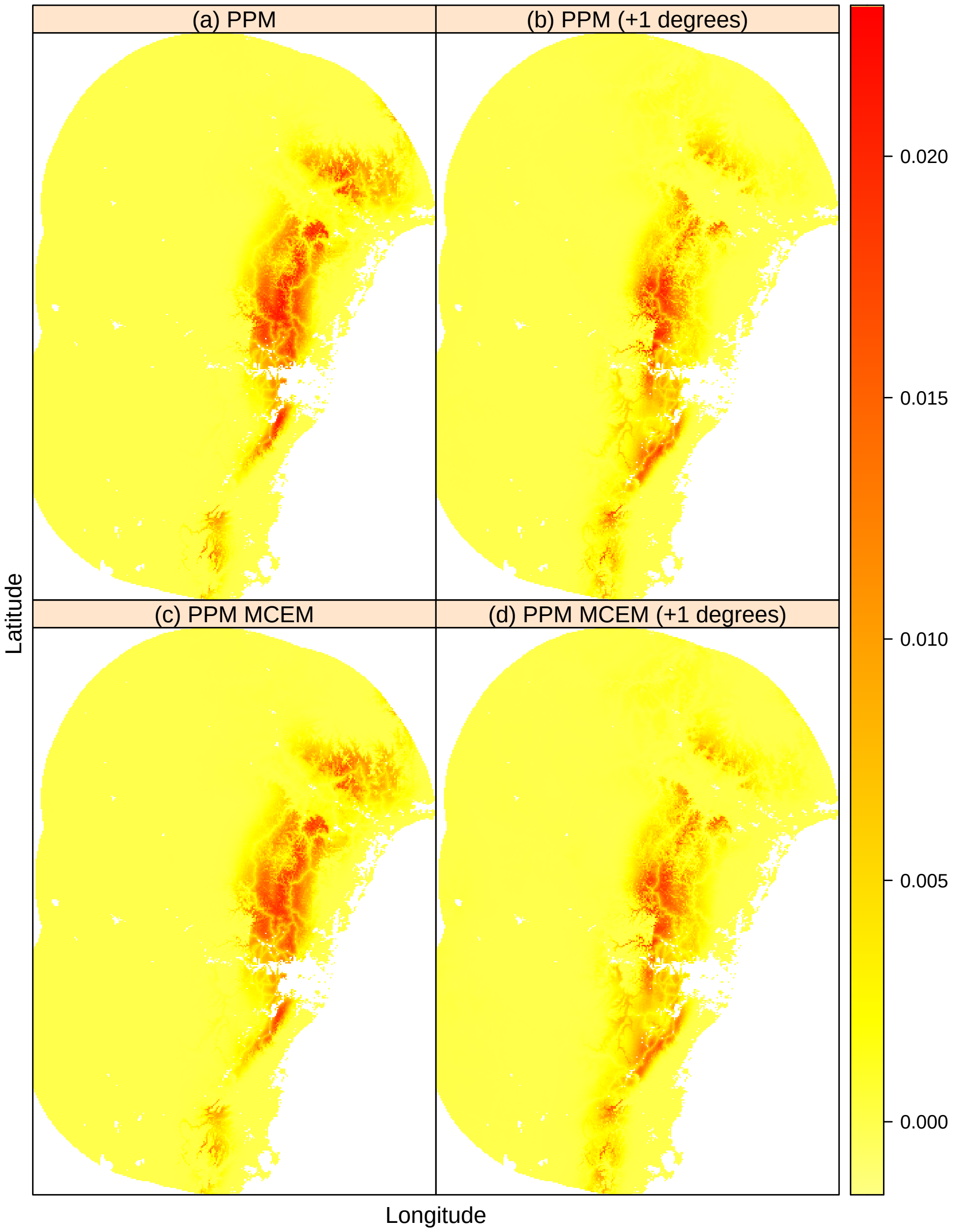

S1.6: *Predicted presences of Corymbia eximia using presence-only data and the max temperature covariate. We assume the temperature covariate is subject to measurement error ($\sigma_u^2 = 0.25$ or with a reliability ratio of 93%). Models in the top row do not account for measurement error in the max temperature variable (reliability ratio of 100%) (a) PPM; (b) PPM with a 1.0 degree shift in max temperature; (c) PPM MCEM; and (d) PPM MCEM with a 1.0 degrees Celsius shift in max temperature.*

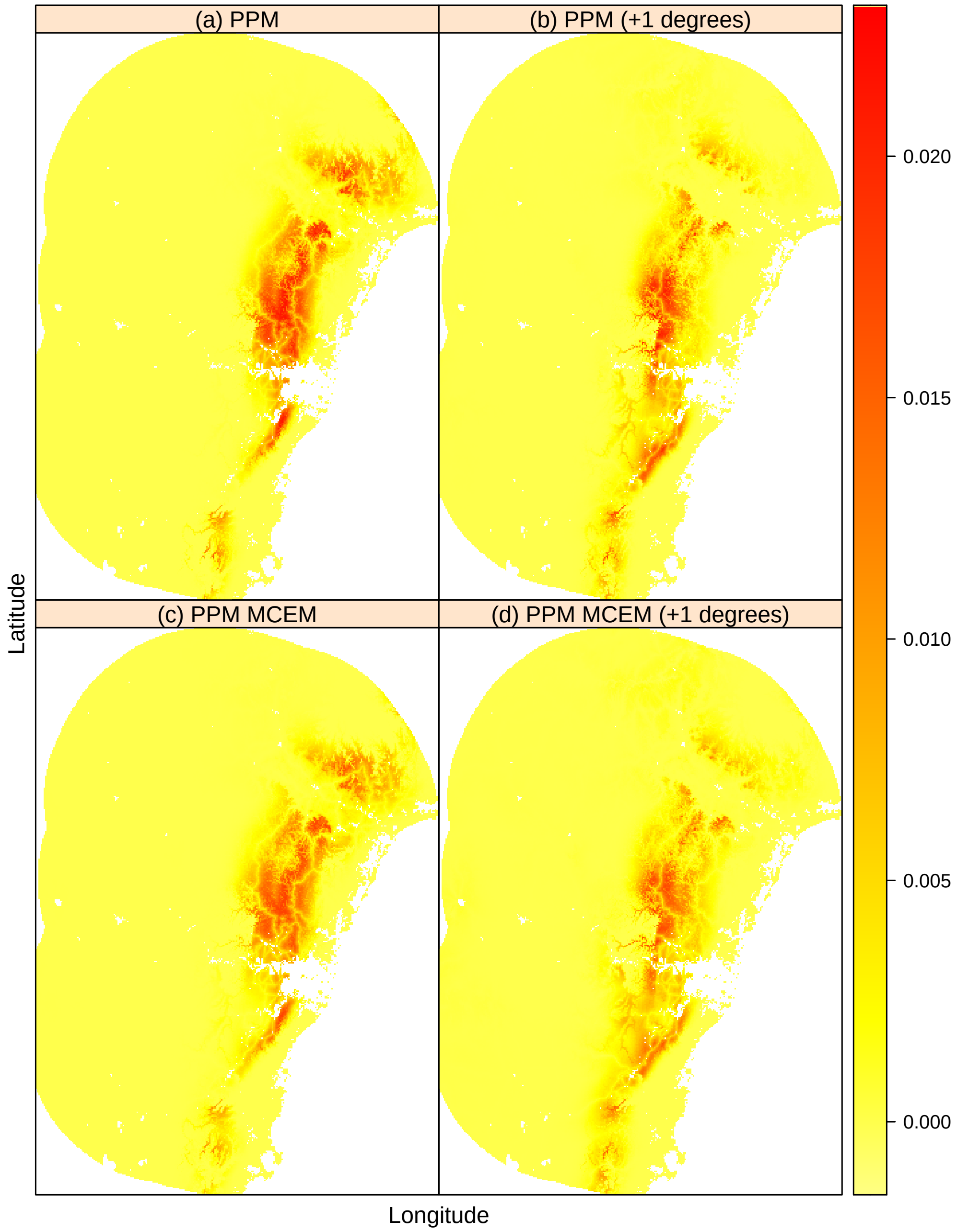

S1.7: *Predicted presences of Corymbia eximia using presence-only data and the max temperature covariate. We assume the temperature covariate is subject to measurement error ($\sigma_u^2 = 0.5$ or with a reliability ratio of 87%). Models in the top row do not account for measurement error in the max temperature variable (reliability ratio of 100%) (a) PPM; (b) PPM with a 1.0 degree shift in max temperature; (c) PPM MCEM; and (d) PPM MCEM with a 1.0 degrees Celsius shift in max temperature.*

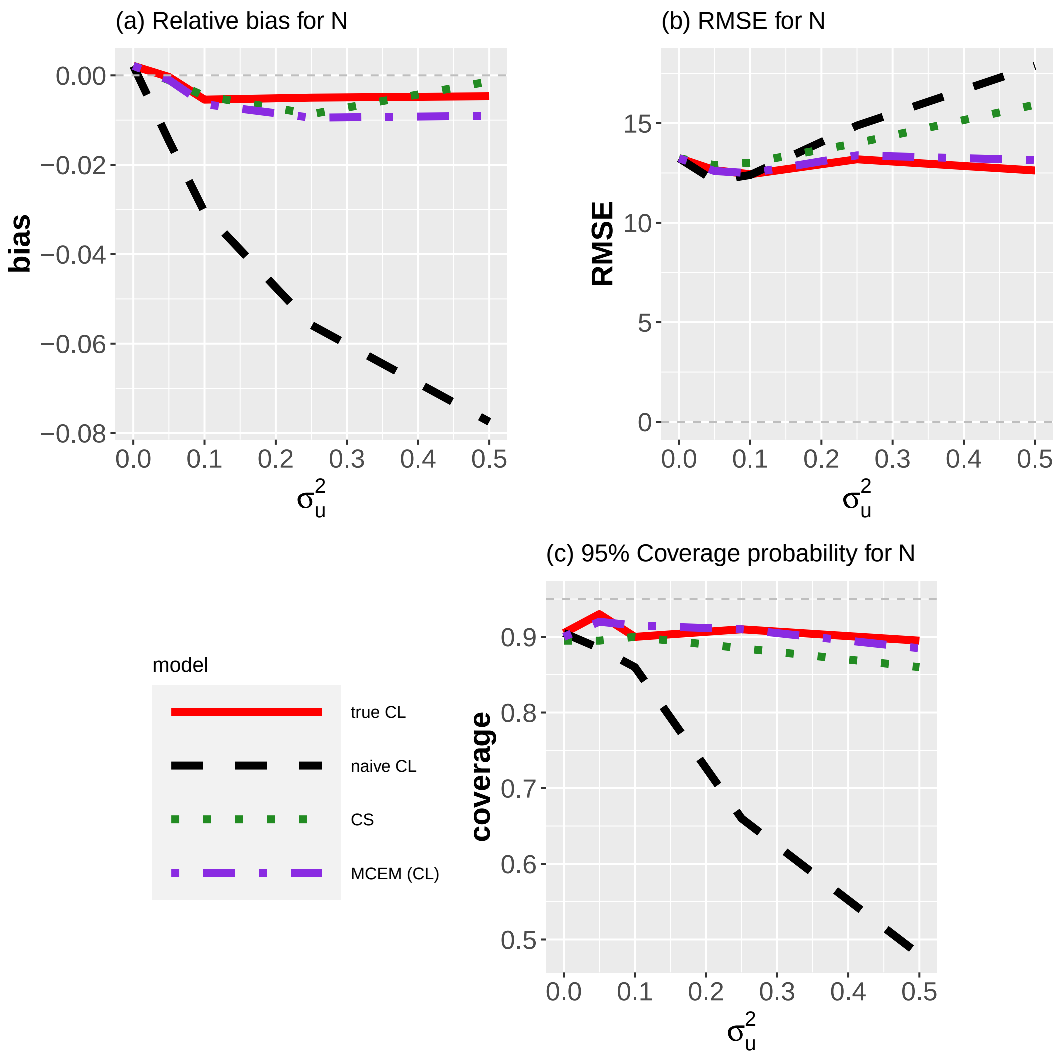

S1.8: *Capture–recapture simulation study, case (i): (a) Relative bias, (b) RMSE and (c) 95% coverage probabilities for N when fitting linear conditional likelihood (CL) models to simulated capture–recapture data with one error-contaminated covariate across increasing measurement error values σ_u^2. We compare results when fitting the true CL model, the naïve CL model, a conditional score (CS) model and MCEM using the CL. The true population size is $N = 200$ and $\tau = 7$.*

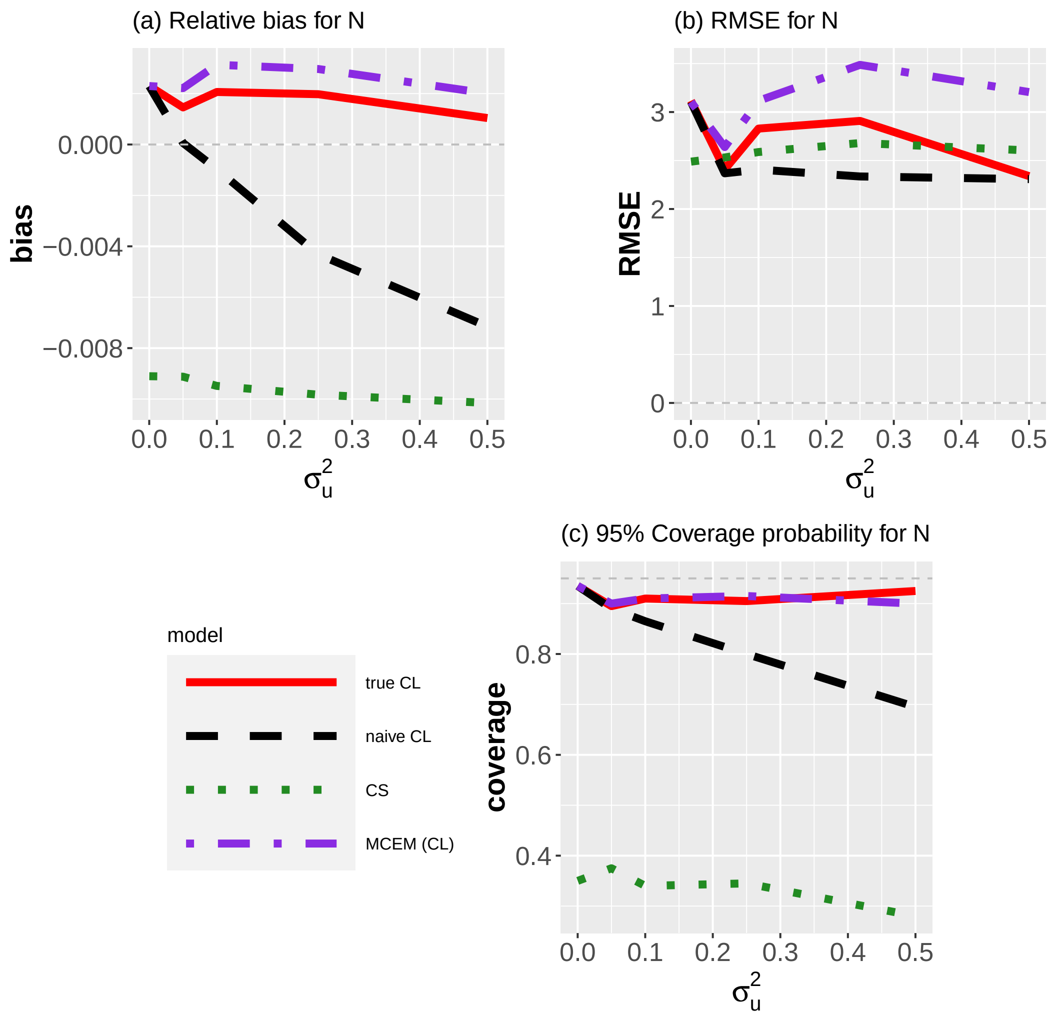

S1.9: *Capture–recapture simulation study, case (ii): (a) Relative bias, (b) RMSE and (c) 95% coverage probabilities for N when fitting quadratic conditional likelihood (CL) models to simulated capture–recapture data with one error-contaminated covariate across increasing measurement error values σ_u^2. We compare results when fitting the true CL model, the naïve CL model, a conditional score (CS) model and MCEM using the CL. The true population size is $N = 200$ and $\tau = 7$.*
